# The Role of Light Irradiation and Dendrimer Generation in Directing Electrostatic Self-Assembly

**DOI:** 10.3390/polym17020170

**Published:** 2025-01-11

**Authors:** Mohit Agarwal, Alexander Zika, Müge Yücel, Ralf Schweins, Joachim Kohlbrecher, Franziska Gröhn

**Affiliations:** 1 Department of Chemistry and Pharmacy, Interdisciplinary Center for Molecular Materials, Friedrich-Alexander Universität Erlangen-Nürnberg, Egerlandstr. 3, 91058 Erlangen, Germany; 2 Institut Laue-Langevin, Large Scale Structures Group, 71 Avenue des Martyrs, F-38000 Grenoble, France; 3 Paul Scherrer Institut PSI, Forschungsstrasse 111, 5232 Villigen, Switzerland

**Keywords:** azo dye, dendrimer, electrostatic self-assembly, light irradiation, photoresponsive, switchability

## Abstract

pH-responsive polyamidoamine (PAMAM) dendrimers are used as well-defined building blocks to design light-switchable nano-assemblies in solution. The complex interplay between the photoresponsive di-anionic azo dye Acid Yellow 38 (AY38) and the cationic PAMAM dendrimers of different generations is presented in this study. Electrostatic self-assembly involving secondary dipole–dipole interactions provides well-defined assemblies within a broad size range (10 nm–1 μm) with various shapes. The size and shape of these assemblies were determined using dynamic and static light scattering (DLS/SLS) and small-angle neutron scattering (SANS); ζ-potential measurements were performed to elucidate the charge characteristics, revealing the effective surface charge density of the nano-objects as an important parameter in the size and shape control. UV–vis spectroscopy and isothermal titration calorimetry (ITC) were employed to investigate the interaction on a molecular level and from a thermodynamic point of view. The results show that the amount of isomerized *cis* dye depends on the dendrimer generation because of a photoprotective effect through electrostatics for lower generations and through dipole–dipole interactions for higher generations; as the *cis* dye and *trans* dye bind with different strength, the amount of *cis* dye then again encodes the charge density and thereby the particle size and shape.

## 1. Introduction

Self-assembly, a feasible and effective process, offers a powerful tool to construct defined nanoparticles via various noncovalent interactions such as π-π stacking [1,2], amphiphilicity [3,4,5], H-bonding [6], and ionic interactions [7,8,9,10]. Electrostatic self-assembly refers to the self-assembly process driven by ionic interactions between components [11,12,13,14,15,16]. These interactions include Coulombic attraction (or repulsion) between charged species, often in conjunction with other non-covalent interactions such as π-π interactions, hydrogen bonding, or dipole–dipole interactions [17,18,19], referred to as secondary interactions as they only come into play when the building blocks approach each other due to their opposite charge. Electrostatic interactions are non-directional; therefore, secondary interactions often play a crucial role in producing desired structures [16]. This approach can be used to produce nano-objects of various shapes, such as spheres, cylinders, disks, and ellipsoids [16]. In particular, electrostatic self-assembly is a powerful technique that is capable of creating nanostructures in aqueous solution that respond to various stimuli, e.g., changes in temperature, pH, concentration, light irradiation, or the presence of certain chemicals [12,19,20,21,22]. This responsiveness can be built into nano-assemblies by using components that respond to the desired stimuli [23,24,25]. Our group demonstrated the electrostatic self-assembly of macroions and stiff multivalent counterions, e.g., dendrimer-dye nano-objects in solution, showcasing how charge interactions govern size and shape [21,26,27].

Photo-driven systems are especially very interesting for altering the nanostructure in aqueous solution [16,28,29]. Classical surfactants capable of UV-induced isomerization or dimerization can form light-responsive micelles [30]. Higashi et al. reported the first light-responsive electrostatically self-assembled nanostructures in 2007, where an anionic azobenzene derivative and a cationic β-sheet peptide formed nanofibers [31]. Upon UV irradiation, azobenzene isomerized to its *cis* state, causing the nanofibers to dissolve, while reassembly occurred in the *trans* state. In 2009, Ghoreishi et al. investigated the interactions between anionic azo dye and the cationic surfactant tetradecyltrimethylammonium bromide (TTAB) [32]. Despite advancements in light-triggered assembly and disassembly, controlling the nano-object size through photo-irradiation in solution had remained challenging, and this was first demonstrated by our group in 2010 using an aqueous system of azo-switchable anionic dyes combined with a cationic dendrimer macroion [12]. Up to then, *trans-cis* isomerization had led to triggerable oligomers or gel formation, but switching the particle size had not been achieved with any association interaction type [33,34]. Later, in 2016, Guo et al. fabricated supramolecular structures responsive to UV light, pH changes, and metal ions, highlighting the combination of azobenzene surfactants and Eu-POMs [35].

The first light-switchable particle shape change of an electrostatically self-assembled nano-object was described by us in 2018, where assemblies of a linear polyelectrolyte and an isomerizable azo dye changed from long fibers to more globular structures upon irradiation [22]. Furthermore, Xie et al. explored light-induced isomerization using azobenzene-functionalized peptides [36], demonstrating how light manipulation can dynamically alter supramolecular architectures by exploiting non-equilibrium conditions. More recently, Fathalla et al. used ionic self-assembly to develop pH-responsive porphyrin-silica nanoparticles [37]. The particles released encapsulated drugs in response to pH changes, leveraging the disruption of ionic interactions at acidic pH to achieve controlled release [15,38,39]. These systems underline the key role of macroions’ behavior in stimuli-responsive materials. Electrostatic self-assembly provides a foundation for the structuring of nanomaterials, where stimuli like light and pH offer tunable mechanisms for dynamic responses. However, structure-directing principles, for the first time, were investigated in our recent study in this journal (based on the dependence on the loading ratio) [19] and in this manuscript (influence of the dendrimer generation).

The properties of such photoresponsive electrostatic self-assembly systems can be controlled by changing the intermolecular interactions between building blocks, leading to structural changes in the material. Willerich et al. previously developed a method where we were able to achieve photoswitchable assemblies with nearly monodisperse nanoparticles using an excess of azo dyes, in which the surplus dye molecules bound by secondary interactions electrostatically stabilized these assemblies through the excess of opposite charges [12]. Later, in a study by Mariani et al., we observed the effects of dendrimer generation on electrostatic assemblies with various (non-switchable) multivalent anionic dye molecules as connecting counterions, finding an increase in shape anisotropy when the dendrimer generations increased in the presence of excess dendrimer [21]. Yet, still missing so far is a study targeting the gain in the fundamental understanding of directing photoswitchable electrostatic self-assembly into nano-objects of various shapes and sizes.

**Scheme 1:**
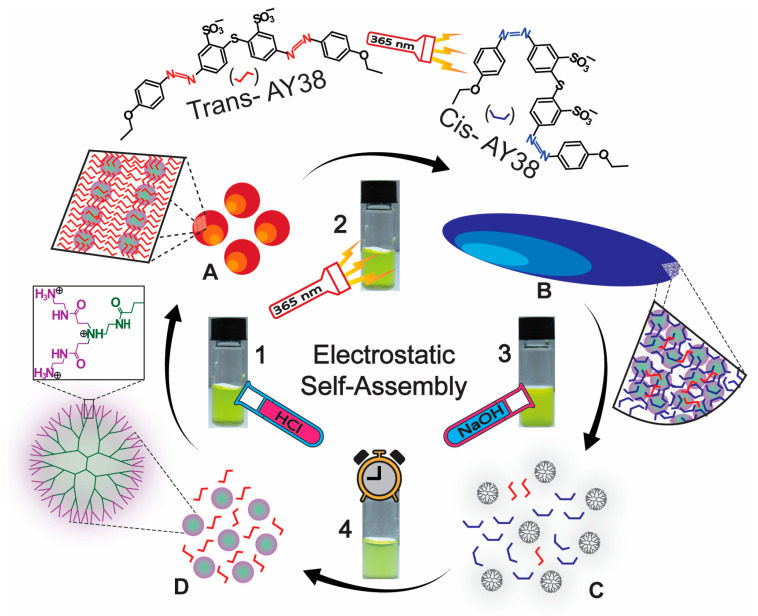
Simplified sketch of the recurring assembly formation at the nanoscale. The molecular structures of the dendrimers and AY38 are shown. A–D represent different nanoassembly structures. Red objects represent simplified *trans* dye molecules, and blue objects indicate *cis* dye molecules. The glass vials carry the same sample solution at different steps of the cycle. A UV lamp with a 365 nm wavelength is used in the experiment.

The present study aims to gain further insight into structure-directing effects and photoresponsiveness by creating and investigating photoswitchable electrostatically self-assembled nano-objects in terms of their dependence on the dendrimer generation. As a model system, a divalent azo dye (AY38, Acid Yellow 38) and cationic polyamidoamine (PAMAM) dendrimers of generation 2 to 8 were employed as the building blocks. The photo-isomerization ability of AY38 and the pH responsiveness of the dendrimers make them suitable candidates for tuning the size and shape of the self-assembled particles by light irradiation and the degree of protonation. In an acidic medium (pH = 3.5), the PAMAM dendrimer contains a maximum number of cationic charges (e.g., 126 charges for the generation 4 dendrimer), where both sets of amino groups (primary end groups and tertiary branching groups) are charged (–NH_3_^+^, –NR_2_H^+^). Here, PAMAM dendrimers are of particular value as model polyelectrolytes, not only because they represent very-well-defined structures but also especially because of their structure change with the generation: early generations of dendrimers exhibit open, star-like morphologies with accessible surface functionalities, while later generations transition into more compact, spherical shapes—still highly swollen with water but with a higher density of organic material inside an overall spherical dendrimer molecule [40,41,42,43]. As the generation increases, the internal part of the dendrimer becomes more “filled”, and the surface functionality becomes increasingly dense, with increased internal crowding and decreased flexibility as well as branching effects constraining diffusion rates or the growth of nanoparticles within the dendrimer [44,45,46]. Various special generation-dependent properties result from this [43]. Overall, with increasing the dendrimer generation, a transition from a typical low-molecular-mass molecule to a typical branched polymer “particle”, or polyelectrolyte in this case, occurs. The AY38 azo dye molecule contains two anionic sulfonate groups (–SO_3_^−^). To build self-assembled nano-structures, the positive amino groups on the dendrimers at pH = 3.5 electrostatically interact with the AY38 dyes, which again are readily isomerized from the *trans* state into the *cis* state by illumination with *λ* = 365 nm. The half-life (*t*_1/2_) of the *cis* state is about 12 h, which is a reasonable timespan to analyze the structural changes due to isomerization. Through the combination of light and pH sensitivity, the electrostatically self-assembled system can undergo recurring assembly (Scheme 1).

Due to the increasing number of branching units and terminal functional groups, higher-generation dendrimers offer more potential binding sites for dye molecules, which could lead to larger nanoparticles in solution. However, the dendrimers become more densely packed with branches, increasing the steric hindrance, making the dendrimer molecules less flexible with increasing the generation. Possibly, this steric hindrance may limit the accessibility of the dye to the internal amino groups, resulting in a lower loading capacity of the dye. In addition, a higher “stiffness” may improve the overall definition and stability of the self-assembled nanoparticles by reducing structural deformations. Lower-generation dendrimers tend to be more flexible and adopt a more extended conformation with a high degree of internal freedom. G0 PAMAM dendrimers, for example, represent “small molecules” and do not form stable assemblies [47]. In contrast, higher-generation dendrimers are less flexible and more constrained, resulting in a stiffer and more compact conformation. Thus, overall, a complex scenario results, guided by several interplaying effects that are yet to be understood.

## 2. Materials and Methods

### 2.1. Chemicals

Polyamidoamine dendrimers with tertiary amino group branches and primary amino groups as surface end groups of generations 2, 3, 4, 5, 6, 7, and 8 were purchased from Aldrich (Darmstadt, Germany). DLS confirmed the radius and size distribution given by the manufacturer Dendritech (Midland, MI, USA), as shown in Figure S1 [48]. Acid Yellow 38 (AY38; >40% purity) was purchased from Merck (Darmstadt, Germany) and purified to >98% by multiple recrystallizations from water and ethanol [49]. D_2_O, NaOD, and DCl were purchased from Merck, Darmstadt, Germany, and NaOD and DCl were used to adjust the pD of the solution.

### 2.2. Sample Preparation

AY38 and PAMAM dendrimer stock solutions were prepared using D_2_O solvent at pD = 10.5 (pD = pH + 0.4) [50]. Both stock solutions were mixed at room temperature while stirring. An apposite amount of DCl was then added to the solution to reach a pH/pD of 3.5 to induce self-assembly. The concentration of AY38 dye was kept constant at *c* = 1.0 × 10^−4^ mol L^−1^ in the majority of the samples throughout the study with varied dendrimer concentrations. In one part of the results, the dendrimer concentrations were kept constant at *c* = 1.0 × 10^−7^ mol L^−1^ and the dye concentration was varied. The samples were prepared according to their charge ratio, *l_c_* (a ratio of the number of anionic dye sulfonate groups to the number of cationic amine groups). A ratio below unity (<1.0) corresponds to an excess of positive dendrimer charges, and a ratio higher than unity (>1.0) corresponds to an excess of negatively charged AY38 molecules.

The sample vials were illuminated with a UV lamp (power = 230 Volts) at *λ* = 365 nm for 20 min while continuously stirring. The AY38 dye readily achieved its photostationary state (≈20 min). Measurements were performed immediately after irradiation.

### 2.3. Light Scattering

Static and dynamic light scattering are highly suitable techniques for characterizing polyelectrolytes in solution. Dynamic light scattering (DLS) probes the temporal fluctuations in the scattered intensity by using coherent laser light. Instead of the scattering angle *θ*, the scattering vector $\vec{q}$ (also known as momentum transfer) is used and is shown as follows [51]:

(1) $$\vec{q}=\vec{k_{s}}-\vec{k_{i}}$$

where $\vec{k_{i}}$ and $\vec{k_{s}}$ are the propagation vectors of the incident and the scattered light. The magnitude of $\vec{q}$ is given by:

(2) $$q=\left|\vec{q}\right|=\frac{4\pi n_{D}}{\lambda }sin\left(\frac{\theta }{2}\right)$$

with *λ* being the wavelength of incident light in the solution, and *n_D_* is the refractive index of the solvent.

The intensity–time autocorrelation function, $g_{2}$(*q*, *τ*), is given by:

(3) $$g_{2}\left(q,\tau \right)=\frac{\langle I\left(q,t\right)I(q,t+\tau )\rangle }{{\langle I\left(q,t\right)\rangle }^{2}}$$

The above function describes the correlation between the scattering intensity at time *t* with the one at time *t* + *τ*, where *τ* is the shift in time. The intensity–time autocorrelation function can be converted into the electric field–time autocorrelation function $g_{1}$(*q*, *τ*) using the Siegert relation:

(4) $$g_{2}\left(q,\tau \right)=1+{\left[g_{1}\left(q,\tau \right)\right]}^{2}$$

The $g_{1}$(*τ*) function was further analyzed by a regularized inverse Laplace transformation using the CONTIN algorithm by S. Provencher to obtain the relaxation time distribution [52,53]. The mean relaxation times were then transferred into the apparent diffusion coefficient *D_app_* using the relation:

(5) $$D_{\mathrm{app}}=q^{-2}\tau^{-1}$$

The measurements covered a broad range of *q* by measuring a scattering angle of 20° ≤ *θ* ≤ 150° with 5-degree steps. In case of an angular dependence, *D_app_* was extrapolated to *q* = 0 to yield *D*_0_. In the absence of an angular dependence, the average value of *D_app_* was taken as *D*_0_. From the latter, the hydrodynamic radius (*R_H_*) can be calculated using the Stokes–Einstein relationship:

(6) $$R_{H}=\frac{kT}{6\pi \eta D_{0}}$$

where *k* is the Boltzmann constant, *T* is the absolute temperature in Kelvin, and η is the solvent viscosity.

Static light scattering (SLS) uses visible light as a characterization probe for particles in solution. In an SLS experiment, the macroscopic scattering cross-section $\frac{d\sum }{d\Omega }\left(q\right)$ is measured and is commonly denoted as the Rayleigh ratio, $\Delta R_{\theta }$:

(7) $$\frac{d\sum }{d\Omega }\left(q\right)=R_{\theta }=KcM_{w}P\left(q\right)$$

where *K* is the optical constant, *c* is the mass concentration, *M_w_* is the weight-averaged molecular weight, and *P*(*q*) is the form factor. The Rayleigh ratio, $\Delta R_{\theta }$, is essentially a measure of the intensity of light scattered by a sample relative to the intensity of the incident light, normalized by the volume of the scattering medium and the distance to the detector. It can be calculated using the formula below:

(8) $$\Delta R_{\theta }=\frac{I_{\theta }r^{2}}{I_{0}V}$$

where $I_{\theta }$ is the scattered intensity at an angle *θ*, with the incident light intensity $I_{0}$, *V* is the scattering volume, and *r* is the distance of the scattering volume to the detector.

The optical constant, *K*, in Equation (7) is given by:

(9) $$K=\frac{4\pi^{2}}{\lambda_{0}^{4}N_{A}}{\left(n_{std}\frac{dn}{dc}\right)}^{2}$$

with *λ*_0_ being the wavelength of the incoming light in a vacuum, *N_A_* is the Avogadro constant, *n_std_* is the refractive index of the standard (toluene) used for calibration of the absolute intensity, and d*n*/d*c* is the refractive index increment (and also equivalent to the scattering length density Δ*ρ* used in SANS experiments). The scattering intensity at low *q* can be approximated by either the Guinier approximation for the form factor *P*(*q*):

(10) $$P\left(q\right)\approx \exp\left(\frac{-q^{2}R_{G}^{2}}{3}\right)$$

or by the Zimm equation [54]:

(11) $$\frac{Kc}{\Delta R_{\theta }}=\frac{1}{M_{w}}\left(1+\frac{1}{3}q^{2}\langle R_{G}^{2}\rangle \right)+2A_{2}c$$

with *R_G_* being the radius of gyration and *A*_2_ being the second osmotic virial coefficient. The Zimm and Guinier equations are only valid for *q* × *R_G_* ≤ 1.2 [55]. The ratio of *R_G_*/*R_H_* provides essential information on the particle topology.

Measurements were carried out using an ALV CGS3 goniometer with an ALV 5004 correlator (ALV, Langen, Germany) equipped with a HeNe laser with a wavelength of *λ* = 632.8 nm and a maximum output power of 23 mW.

### 2.4. Small-Angle Neutron Scattering

Polymers and polyelectrolytes are typically analyzed in solution and have a low contrast for electron microscopy and small-angle X-ray scattering (SAXS). Since the neutron scattering length *b* depends on the isotope and differs significantly for hydrogen and deuterium, it is advantageous to use deuterated compounds or solvents to achieve better contrast with neutrons. Here, SANS measurements were carried out using the D11 instrument (https://doi.ill.fr/10.5291/ILL-DATA.9-12-662, accessed on 30 November 2024) at the Institut Laue Langevin (ILL) in Grenoble, France, and using the SANS-I instrument (Proposal 20221454) in PSI, Villigen, Switzerland.

The SANS samples were prepared in D_2_O with an AY38 concentration of *c* = 0.07 gL^−1^ and transferred into Hellma 404-QX quartz cells with a 2 mm path length.

At the ILL, the irradiated samples with different PAMAM generations (G2 to G8) were measured. For this, a particular setup was used, where the employed neutron beam had a diameter of 15 mm. A neutron wavelength *λ* = 6.0 Å (FWHM 9%) with three different sample–detector distances (1.4 m, 8 m, and 38 m) with collimation distances of 8 m, 8 m, and 40.5 m were used, respectively. In total, a scattering vector range of 0.016 nm^−1^ ≤ *q* ≤ 4.5 nm^−1^ was investigated.

Scattered neutrons were detected using a multitube ^3^He gas detector consisting of three detector panels: a central one with 192 horizontally oriented tubes and a left- and right-side panel of 32 vertically oriented tubes. Each tube was 1 m long, with a pixel size of 4 mm along the tube, with the inner tube diameter being 8 mm. The detector was, therefore, composed of 256 × 256 pixels. Two-dimensional data were corrected for empty cell scattering, electronic background, detector uniformity via measuring 1 mm H_2_O scattering, and transmissions using the Mantid 6.0 software package [56]. Data were put on an absolute scale via the attenuated direct beam intensity, measured per instrument configuration. Azimuthally averaged scattering curves were then used to subtract the solvent scattering and incoherent background.

The SANS *q*-range was extended in the low-q region by 0.0046 nm^−1^ ≤ *q* ≤ 0.025 nm^−1^ using SLS data. SLS data were merged with SANS data to extend the low-*q* range to obtain a more reliable insight into the shape and size of the nanoparticles. The extended low-*q* range allows for the coverage of the dimensions of larger aggregates. The exact same samples were measured with both techniques.

Parts of the samples were measured using the PSI SANS-I instrument with a different setup, where the experiments were carried out at 3 different sample-to-detector distances (1.6 m, 6 m, and 18 m) with collimation distances of 6 m, 6 m, and 18 m, respectively. A neutron wavelength of *λ* = 6 Å was used to cover a total q range of 0.03 nm^−1^ ≤ *q* ≤ 4.0 nm^−1^. The circular diameter of the employed neutron beam was 14 mm. The scattered neutrons were detected using a 2D MWPC CERCA ^3^He detector with 128 × 128 elements of 7.5 × 7.5 mm^2^. Similar to the experiments at the ILL, the detector images were corrected using a water sample, and the BerSANS (2002) software was used to correct transmissions and to azimuthally average the samples. All the measurements were performed at room temperature (25 °C). In the end, the solvent scattering and the incoherent background were subtracted from the samples’ scattering curves.

The shapes and dimensions of the nanoparticles were determined by fitting the data with appropriate structural models using the SasView 4.2.2 software. The SasView 4.2.2 software fits the scattering data using various model form factors.

The solvent scattering length density was known and fixed while fitting. The reduced *χ*^2^ values were used to determine the quality of the SANS data fit. All the fitted models were well fit, as observed by their *χ*^2^ value (≤3.0).

The SANS intensity after data correction is as follows [57]:

(12) $$I\left(q\right)=\emptyset V_{NP}{\left(\Delta \rho_{SLD}\right)}^{2}P\left(q\right)S\left(q\right)$$

where ∅ is the volume fraction, *V_NP_* is the particle volume, Δ*ρ_SLD_* is the difference in scattering length densities between solute and the solvent, and *P*(*q*) is the particle form factor corresponding to the particle shape. The structure factor *S*(*q*) represents the interparticle correlations and was considered to be equal to 1, as the samples were diluted such that no interparticle interactions were present. The form factor is the square of the normalized total scattering amplitude *F*(*q*).

For example, for a sphere with radius *R*, the scattering amplitude *F*(*q*) is [55]:

(13) $$\;F\left(q\right)=3\frac{sinqR-qRcosqR}{{\left(qR\right)}^{3}}$$

For a cylinder with length *L* and radius *R*, *P*(*q*) is [58,59]:

(14) $$P\left(q,\alpha \right)=\frac{scale}{V}F^{2}\left(q,\alpha \right)\sin\left(\alpha \right)+background$$

where

(15) $$F\left(q,\alpha \right)=2\left(\Delta \rho \right)V\frac{sin\left(\frac{1}{2}qLcos\alpha \right)}{\frac{1}{2}qLcos\alpha }\frac{J_{1}\left(qRsin\alpha \right)}{qRsin\alpha }$$

where *α* is the angle between the cylinder’s axis, *V* = *πR*^2^*L* is the volume of the cylinder, Δ*ρ* (contrast) is the scattering length density difference between the scatterer and the solvent, and *J*_1_ is the first-order Bessel function.

For an ellipsoid, the scattering amplitude *F*(*q*) is [60]:

(16) $$F\left(q,\alpha \right)=\Delta \rho V\frac{3(\sin qr-qr\;cos\;qr)}{{\left(qr\right)}^{3}}$$

where

(17) $$r={\left[R_{e}^{2}sin^{2}\alpha +R_{p}^{2}cos\alpha \right]}^{1/2}$$

where *α* is the angle between the axis of the ellipsoid, $V=\left(4/3\right)\pi R_{p}R_{e}^{2}$ is the volume of the ellipsoid, *R_p_* is the polar radius along the rotational axis of the ellipsoid, and *R_e_* is the equatorial radius perpendicular to the rotational axis of the ellipsoid.

### 2.5. ζ-Potential Measurements

The particles and their microenvironment determine the electrostatic properties of nanostructures in a solution. Such properties of nanostructures can be characterized by ζ-potential measurements. This technique measures the difference in the electrostatic potential between the medium and the fluid layer of the dispersed nanostructures. The ζ-potential was calculated from the electrophoretic mobility using the Helmholtz–Smoluchowski equation [61,62]:

(18) $$\zeta =\frac{\mu_{e}\eta }{\varepsilon_{rs}\varepsilon_{0}}$$

where *μ_e_* is the electrophoretic mobility, η is the dynamic viscosity of the liquid, *ε_rs_* is the dielectric constant of the electrolyte solution, and *ε*_0_ is the electric permittivity of the vacuum.

The ζ-potential measurements were conducted on a Zetasizer Nano ZS analyzer with an integrated 4 mW HeNe laser, with *λ* = 633 nm (Malvern Instruments Ltd., Malvern, UK).

### 2.6. UV–vis Spectroscopy

The absorption spectra were recorded with a JASCO V-630 spectrometer using quartz cuvettes with a 1 mm path length. Each measurement was performed at room temperature. UV–vis spectroscopy was used for determining the amount of dye in the solution and the dye involved in the nanoassemblies by combining it with centrifugation and calculating the dye concentration from the UV–vis spectra. In this method, centrifugation can remove all the aggregates (which will also remove all the bound dye) and only unbound dye molecules will remain in the solution.

### 2.7. Isothermal Titration Calorimetry (ITC)

This work used the platforms of the Grenoble Instruct-ERIC center (ISBG; UAR 3518 CNRS-CEA-UGA-EMBL) within the Grenoble Partnership for Structural Biology (PSB), supported by FRISBI (ANR-10-INBS-0005-02) and GRAL, financed within the University Grenoble Alpes graduate school (Ecoles Universitaires de Recherche) CBH-EUR-GS (ANR-17-EURE-0003). ITC experiments were performed at the VP-ITC microcalorimeter.

ITC is a technique that measures the heat changes (released or absorbed) when two solutions are mixed. Two identical reference and sample cells, with a volume of 1.442 mL, were enclosed in an adiabatic jacket. In this study, the reference cell was filled with the solvent (D_2_O), and the working cell contained the sample solution. A 288 μL volume of AY38 azo dye (titrant) from the syringe was injected stepwise into the sample cell containing the dendrimer solution under constant stirring. All measurements were carried out at 25 °C. Small aliquots of titrant (typically 2 μL) were injected in 50–60 steps to achieve a charge ratio range of 0.2 ≤ *I_c_* ≤ 4.4. The duration of each injection was 300 s, with a total measurement time of 4–4.5 h (including the equilibration time). Due to the absorption or release of heat, each injection produced a distinct peak. A separate measurement was performed with the titrant and the solvent for data subtraction. Data analysis and fitting were performed using the MicroCal Origin 7.0 software. The data were fitted using the two-site model. The Gibbs free energy (Δ*G*) is calculated using the following:

(19) ΔG=ΔH−TΔS=−RTlnK

## 3. Results

This study focused on developing a system of self-assembled nanostructures with a light-tunable particle size and shape in a wide size range (10 nm–2 µm). The structural characterization was based on static and dynamic light scattering and small-angle neutron scattering analyses to investigate the size and shape of the nanoparticles. Systematic surface potential studies investigated the cause of the stability of the nanoparticles in an aqueous solution. UV-vis and ITC measurements were performed to analyze the interactions at a molecular level and from a thermodynamic perspective, respectively.

Scheme 1 represents the four assembly steps, where step 1 creates the nanoassemblies before irradiation in the acidic medium (pD = 3.5). Step 2 occurred after the irradiation. Step 3 and step 4 were carried out to bring the sample into its initial stage by adding NaOD (until pD = 11.5) and DCl (until pD = 3.5) sequentially. The cycle could be reproduced multiple times without any major influence on the assembly structures.

### 3.1. Structural Characterization

#### 3.1.1. Light Scattering: Size Distributions Before and After Irradiation

**Figure 1:**
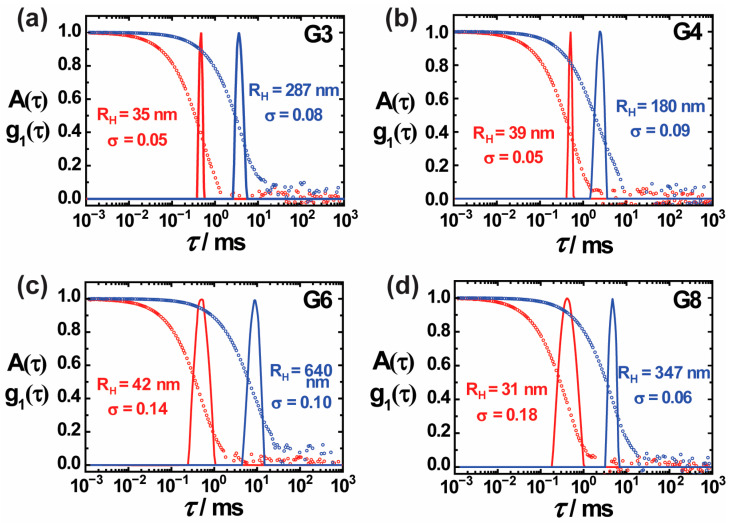
Multi-angle DLS at a charge ratio (dye charges/dendrimer charges) of *l_c_* = 1.5 before (red) and after irradiation (blue): electric field autocorrelation *g*_1_(*τ*) (open circular symbols) and distribution of relaxation times A(*τ*) (lines) at a scattering angle of *θ* = 90° for different dendrimer generations: (**a**) G3, (**b**) G4, (**c**) G6, and (**d**) G8. (The *R_H_* values were obtained by extrapolation to a 0° angle, and σ represents the particle size distribution at a 90° scattering angle, c_AY38_ = 1 × 10^−4^ mol L^−1^.)

Figure 1 illustrates the DLS electric field autocorrelation functions *g*_1_(*τ*) for the assemblies of AY38 with four different dendrimer generations: G3, G4, G6, and G8 (all *c*_AY38_ = 1 × 10^−4^ mol L^−1^) with their corresponding distribution of relaxation times A(*τ*) for a scattering angle of *θ* = 90°. The DLS results of the other dendrimer generations (G2, G5, and G7) are shown in Figure S2. The hydrodynamic radius, *R_H_*, was calculated by extrapolating the angular-dependent diffusion coefficient values to a zero-degree angle and using the Stokes–Einstein relationship (Equation (6)). The *σ*-value, the standard deviation, represents the polydispersity of the nanoparticles in the solution, which is the average polydispersity of all the scattering angles measured (20–150°). Before irradiation, the hydrodynamic radii of the different nanostructures were found to be in the range of 31 nm ≤ *R_H_* ≤ 42 nm with a low polydispersity of 0.05 ≤ *σ* ≤ 0.18, where the aggregate size increased up to G6 (*R_H_* = 42 nm) and decreased again in the higher-generation assemblies. Interestingly, the generation dependency was opposite to the outcome of the study of Mariani et al., where higher generations formed larger aggregates [21]. Such a stark difference was likely due to the nature of the dye–dendrimer and/or dye–dye interactions, as a different azo dye (AY38) was used in the present study, as will be further discussed below. Upon irradiation, the nanoparticles reassembled to form larger (up to 15-fold) and highly monodisperse (0.06 ≤ σ ≤ 0.10) assemblies. Overall, the light scattering results (before and after irradiation) were consistent with a previous proof-of-concept study, in which photoresponsive AY38/G4 assemblies with a similar size range were found [12].

Dynamic light scattering (DLS) measurements at a scattering angle of *θ* = 90° revealed monomodal assemblies, as shown in Figure 1. However, at lower angles (*θ* ≤ 70°), a small intensity-weighted peak (<5%) corresponding to larger particles (≈1 µm) was observed for the high-generation dendrimers (≥G6), as shown in Figure S3. The effects of the multi-angle scattering are further illustrated in Figure S4, where bimodal particle size distributions were detected at small angles (*θ* < 90°), while only single particle size distributions were observed at larger angles (*θ* ≥ 90°). The diffusion coefficient, *D*, is also shown as a function of the square of the scattering vector, *q*^2^, for the monomodal (AY38-G3) assemblies in Figure S5 and the bimodal assemblies (AY38-G7) in Figure S6. These observations align with the principles of light scattering: the detection angle is inversely related to the particle size, meaning larger particles are more easily detected at smaller angles. Thus, it is plausible that “very large” aggregates are exclusively observed at smaller angles.

Evidently, the dendrimer generation plays a crucial role in the structure formation. These large aggregates were likely formed in the high-generation dendrimers due to their reduced flexibility and significant steric hindrance. This steric constraint may limit the extent of the interaction between the dye ions and the charged functional groups on the dendrimers, leaving a portion of dye ions binding via secondary dipole–dipole interactions to electrostatically bound dye ions. This interaction was proposed as the primary mechanism inducing bimodality in the electrostatic self-assemblies [19] and will be elucidated in the following sections using complementary analytical methods.

**Figure 2:**
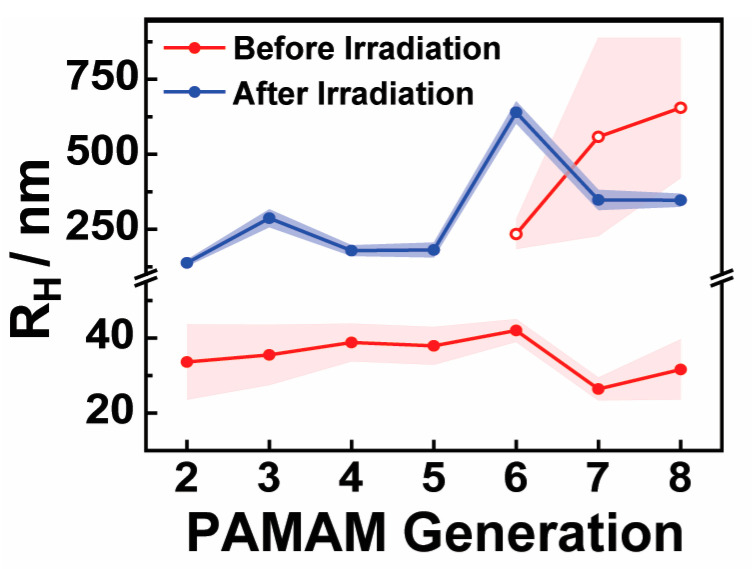
Effect of the irradiation on the hydrodynamic radius *R_H_* for various dendrimer generations at a charge ratio of *l_c_* = 1.5 and constant dendrimer mass concentration. Results are from multi-angle DLS. The shaded areas in blue and red color represent the polydispersity in particle size Three open-circle data points in red represent a second size species in particles for samples with high-generation dendrimers.

Figure 2 represents the overall structural change in the nano-assemblies found through multi-angle DLS at a charge ratio of *l_c_* = 1.5. Previously, the PAMAM generation 2 dendrimer had not been able to form stable electrostatic assemblies, as it had only been explored in solutions with a dendrimer excess [47]. By extension, in the present study, G2 could form stable particles with an excess of AY38 azo dye, though with a large polydispersity (see Figure S2). The surplus dye—bound through secondary dipole-dipole interactions to the electrostatically bound dye molecules (see below)—provides extra charges and, ultimately, extra electrostatic stability to the aggregates. Hence, stabilizing structures with G2 dendrimer might be possible here due to the nature of AY38 azo dye that yields additional stability due to two anionic groups and secondary interactions. However, the larger polydispersity and limited charge ratio range in which stable nano-assemblies can be built with the G2 dendrimer demonstrate that macromolecular—nano-scale—building blocks rather than small-molecular building blocks are required for a versatile and defined structure formation by this type of electrostatic self-assembly.

Furthermore, higher-generation dendrimers were explored, which exhibited highly stable assemblies in the solution with a low polydispersity. Before irradiation, the assemblies did not show any significant size change upon increasing the dendrimer generation ≤ G6, but there was a slight decrease in size from G6 to G7 (which is opposite to what was observed by Mariani et al. [21]). The G8 dendrimer seems to form slightly larger nanoparticles than G7 but with a higher polydispersity, which, therefore, is not considered a significant change here. This remarkable dependence on the generation with a maximum and minimum size and generation 6 and 7, respectively, indicates a complex interplay of effects. Higher-generation dendrimers have a greater number of functional groups, i.e., they are densely populated with cationic amine charges. When these dendrimers interact with anionic AY38 azo dye molecules, the resulting dendrimer-dye complexes can exhibit enhanced electrostatic repulsion due to the high number of charged groups. According to previous studies by us, the assembly size (aggregation number) increases with decreasing the free enthalpy ΔG of the association, which would mean larger aggregates for more highly charged building blocks, i.e., for higher-generation dendrimers [47]. On the other hand, the observation for higher generations may be attributed to their denser structure and decreased flexibility causing the functional groups to be less accessible for the attachment of dye molecule stacks, yielding smaller particles with G7 and G8.

Upon light irradiation, the smaller assemblies, along with a fraction of the larger aggregates observed in the higher-generation dendrimers (as shown in Figure 2), for all dendrimer generations reorganized into larger monomodal assemblies, consistent with observations from previous studies [19]. The increased monodispersity is evident from the reduction in the shaded area of the blue data points following irradiation. This phenomenon arises from the photoisomerization of the azo dye to its *cis* state, which interacts differently with dendrimer molecules compared to the *trans* state due to its altered conformation. This caused the system to reconfigure, forming larger aggregates, ultimately stabilizing the nanoparticles in the solution. Specifically, the *cis* molecule showed a lower dipole moment, resulting in less expressed secondary dipole–dipole interactions [12]. The connection of this molecular effect with the particle size will be discussed further below.

The low-generation dendrimers are small in size, highly flexible, and exhibit a small number of functional groups, resulting in only weakly sterically hindered structures; therefore (similar to before irradiation), the size of the assemblies within the early-generation dendrimers (≤G5) did not differ significantly in terms of the dependence on the generation after irradiation. However, with the G6 dendrimer upon irradiation, the azo dye formed the largest aggregates, which again decreased in size with the higher dendrimer generations. In the case of the G6 dendrimer, there must be a balance of flexibility and steric hindrance, leading to the most favorable conditions for extended particle aggregation. This will be further discussed in detail in conjunction with the UV–vis and ITC studies below.

**Figure 3:**
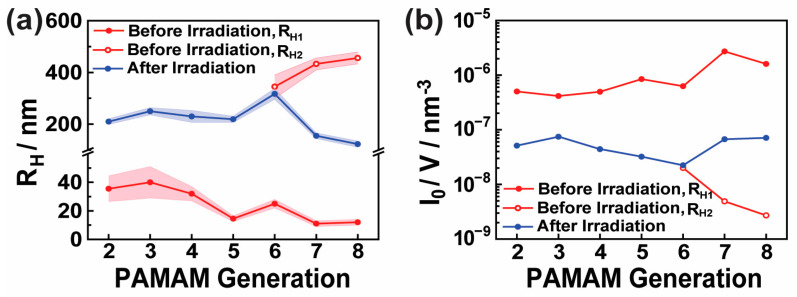
Generation dependence of the dendrimer-dye assemblies at a constant dendrimer number concentration (*c* = 1.0 × 10^−7^ mol L^−1^) for a charge ratio *l_c_* = 1.5 (**a**) Hydrodynamic radius *R_H_* as a function of the dendrimer generation. The shaded areas in blue and red color represent the polydispersity of the particle size. (**b**) Number concentration of the assemblies (approximated using the ratio of the total scattering intensity and the volume (4/3 π *R_H_*^3^) of the assemblies) as a function of the dendrimer generation. The open red symbols represent the second species of the bimodal distributions.

For the results in Figure 1 and Figure 2, the concentration of AY38 was kept constant at *c* = 1.0 × 10^−4^ mol L^−1^. However, to further illustrate the role of the dendrimer generation in the size and shape control, in a further set of experiments, the molar dendrimer concentration was set to *c* = 1.0 × 10^−7^ mol L^−1^, such that the number of dendrimer molecules was identical in the samples with different generations. A moderate excess of dye charges was again used. The results are shown in Figure 3. Figure 3a represents the size of the assemblies at *l_c_* = 1.5, showing a similar trend in sizes to that noticed above in the case of constant dye concentrations. Bimodality was also detected for the higher dendrimer generations (≥G6). For a more detailed understanding, generation-dependent samples with *l_c_* = 2.0 were also investigated (Figure S7a). In the case of *l_c_* = 2.0, bimodality was encountered even earlier, that is, from generation G5 onwards, which was likely due to the higher excess of dye molecules at higher charge ratios, which increased the possibility of secondary dye–dye interactions. This again supports the direct involvement of dye-dye interactions in the control of the polydispersity of the nanoassemblies [19], which will be discussed further alongside the ζ-potential studies below.

If all the dendrimer generations showed similar interactions within the assemblies (regardless of their size), the number of assemblies in the solution would not change, and the number of dendrimer molecules per aggregate would remain the same. Figure 3b depicts the relative number concentration of the assembled nanoparticles as a function of the generation before and after irradiation. This was estimated by dividing the total scattering intensity (*I*_0_) obtained from the extrapolation of the 1*/*Δ*R_θ_* values to a 0° angle (*q* = 0) by the volume of the assemblies approximated as the volume of a sphere, $V=\frac{4}{3}\pi R_{H}^{3}$. It becomes evident that there is an influence of the dendrimer generation on the assembly number concentration, both before and after irradiation.

Before irradiation, a similar trend to when the dye concentration was kept constant in *I*_0_/*V* was observed up to G6, yet increased relative assembly number concentrations were found for the G7 and G8 dendrimers. As a constant molar dendrimer concentration (*c* = 1.0 × 10^−7^ mol L^−1^) was used throughout the samples with different generations, the same number of dendrimer molecules was present in the solution. As the size of the dendrimer increased with increasing the generation, there were fewer dendrimer molecules in the assembly with increasing the generation, as the assembly size was comparable (assuming a similar density). However, in the case of the G7 and G8 dendrimers, where the dendrimer sizes were the largest, smaller assemblies formed, as seen in the DLS studies (Figure 2 and Figure 3a). This led to a higher number concentration of the assemblies for the higher dendrimer-generations. The value of *I*_0_/*V* was the lowest in the samples with two distinct species before irradiation for two significant reasons. First, the low-intensity weighted peak observed for the large particles in the DLS measurements (Figure S3) belonged to the very low *I*_0_ values. Second, the majority of the dendrimer molecules were consumed in the smaller assemblies before irradiation, leaving only a few polyelectrolytes to form the larger aggregates. Upon irradiation, the larger nanoassemblies involved a higher number of dendrimer molecules per particle, which decreased the overall number of particles in the solution. Therefore, the number concentration decreased significantly after irradiation. With the G6 dendrimer, the value observed for *I*_0_/*V* was the lowest. This was because the largest assembly size formed with the G6 dendrimer. Again, a sharp increase in the number concentration was observed in the >G6 generations due to similar reasons as those mentioned above. Overall, interestingly, this consideration showed similar trends to the experiments with a constant mass concentration, which, in return, confirms that the dependencies observed for the experiments with a constant mass concentration were not just an effect of the different number concentration but indeed originated in the dendrimer structure.

#### 3.1.2. LS-SANS: Assembly Shapes Before and After Irradiation

**Figure 4:**
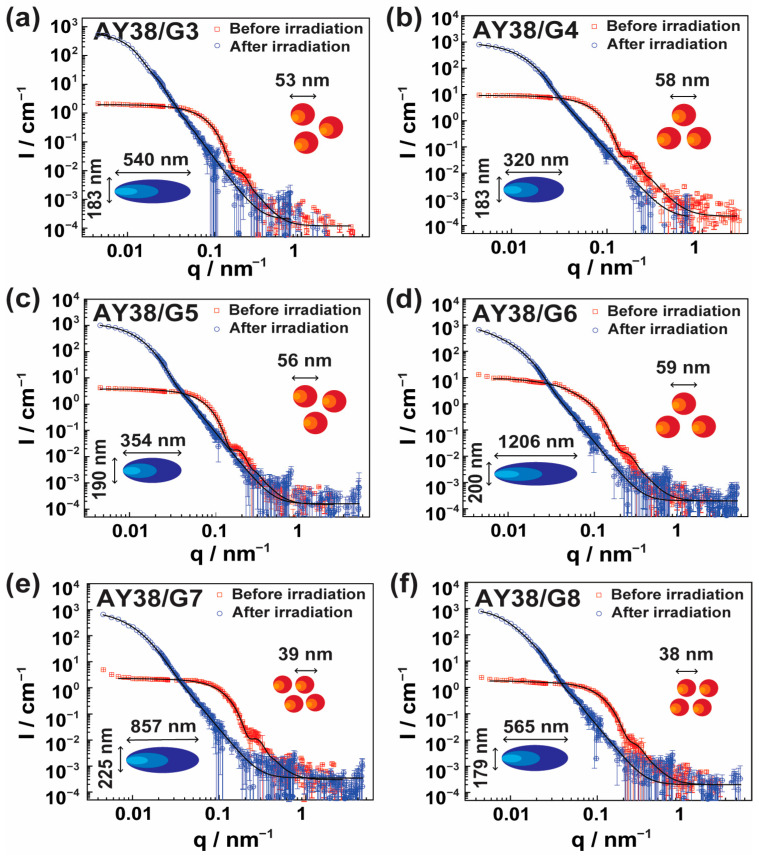
SLS-SANS particle shape analysis: (**a**) AY38-G3; (**b**) AY38-G4, (**c**) AY38-G5, (**d**) AY38-G6; (**e**) AY38-G7, and (**f**) AY38-G8, before (red) and after (blue) irradiation at *l_c_* = 1.5, with data points (symbols) and structural fits (lines). The inserts indicate the particle structures resulting from the structural fitting. Detailed parameters are given in Table S2.

Small-angle neutron scattering was performed to gain insights into the effects of photoirradiation and the dendrimer generation on the shape and dimensions of the self-assembled nanoparticles in solution. The dependency of the size and shape of the assemblies with a constant dye concentration of 1 × 10^−4^ mol L^−1^, i.e., a constant dendrimer mass concentration, on the dendrimer generation, G3 through G8, and photoirradiation is illustrated in Figure 4, and the data for G2 are shown in Figure S8.

Prior to irradiation, spherical particles were observed for the assemblies of all the dendrimer generations except G2. With increasing the generation from G3 to G6, the particle sizes were found to be within the range of 53–59 nm, and smaller assemblies were observed for G7 (39 nm) and G8 (38 nm), which was in accordance with the light scattering results. In some cases, a few data points in the low-*q* region (Figure 4d–f) were not included in the fitting. This observation was consistent with the bimodal particle size distribution of 40–60 nm and >500 nm found in the dynamic light scattering experiment, but the larger assemblies were in a very low quantity. The larger assemblies only started to significantly appear in the low-*q* range obtained from the SLS data. Due to insufficient data points, it was not possible to fit the larger assemblies. Since the scattering vector $\vec{q}$ is inversely proportional to the real space size of the particles (Equation (2)), the low *q*-range from the SANS data did not show any hints of larger assemblies, and they only appeared in the SLS data at very low *q* (*q* ≤ 0.006 nm^−1^). In the case of the G2 dendrimer, small cylindrical nanoparticles were observed (Figure S8). This must be due to the small size of the G2 dendrimer molecules, which evidently affected the overall shape of the assemblies.

**Figure 5:**
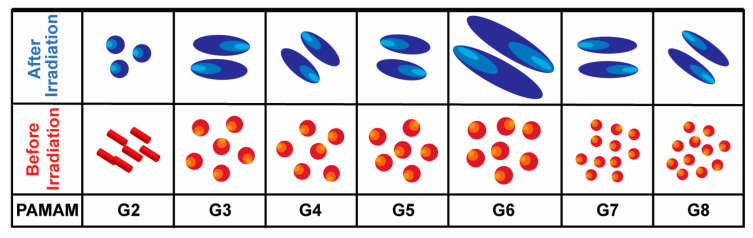
Nanoparticle shapes of dendrimer-dye assemblies with different dendrimer generations obtained from SLS-SANS experiments (the sizes shown in the figure are not scaled.).

After irradiating the samples for 20 min, SANS measurements were carried out, showing that except for G2, all the dye-dendrimer aggregates rearranged into defined ellipsoidal structures. In the case of the G6 dendrimer, the dye-dendrimer interactions resulted in the largest ellipsoid upon irradiation, again in good agreement with the light scattering analysis. The shapes obtained from the SLS-SANS data are summarized in Figure 5.

By combining the information on the particle shape and size, the particle volume—and, consequently, the aggregation number of dendrimers per particle—could be determined from the SLS-SANS analysis more exactly than from the DLS analysis above. This was achieved by dividing the nanoparticle volume by the combined volume of a single dendrimer molecule plus a dye “shell”, as illustrated in Figure S9. The aggregation number decreased with increasing the dendrimer generation, confirming the relative approximation from the DLS analysis, again showing that the size of the dendrimer molecule directly influenced the aggregation number. For instance, the aggregation number (prior to irradiation) decreased from approximately 10^4^ for the G2 dendrimer assemblies to about 10^1^ for the G8 dendrimer assemblies (Figure S9a). However, as shown in Figure S1, the hydrodynamic radius of the G8 dendrimer (5.6 nm) was only 3–4 times larger than that of the G2 dendrimer (*R_H_* = 1.6 nm). This result again suggests that beyond the dendrimer size, steric hindrance played a significant role in limiting the ability of additional dendrimer molecules to interact and form larger aggregates. Upon irradiation, the aggregation number increased, as discussed above.

### 3.2. Charge Characteristics Before and After Irradiation

**Figure 6:**
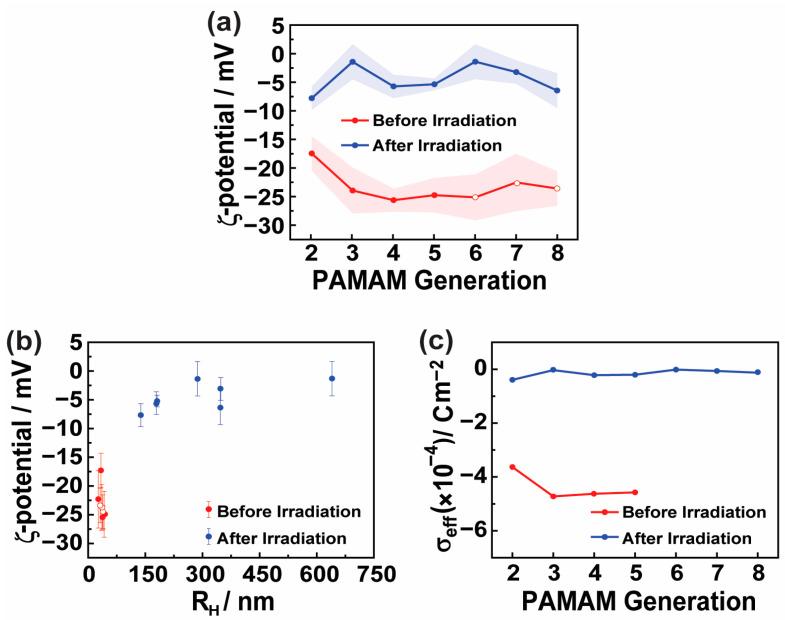
Charge characteristics of dendrimer–dye assemblies: ζ-potential as a function of the (**a**) dendrimer generation and (**b**) hydrodynamic radius, *R_H_*, before and after irradiation. Open red circles represent the biomodal samples. (**c**) Effective surface charge density, *σ*_eff_, of the monomodal assemblies as function of the dendrimer generation. The shaded area in blue and red color in (**a**) shows the error bar of the ζ-potential values.

To obtain a comprehensive understanding of the structure, ζ-potential analysis was conducted to evaluate the influence of the dendrimer generation on the charge status of the assemblies in solution. The results are given in Figure 6. The samples exhibited slightly negative ζ-potential values within the range from −17 to −25 mV, characteristic of well-stabilized assemblies [63]. For the samples with bimodal size distributions (observed prior to irradiation at dendrimer generations ≥G6), the ζ-potential values represented an average across the two particle populations. The ζ-potential contribution from these bimodal distributions is denoted by open red symbols in Figure 6a and should not be interpreted quantitatively.

Upon irradiation, the ζ-potential values increased, shifting closer to 0 mV, indicating a reduction in the electrostatic charge of the particles. It is likely that a decrease in the charge status caused by the molecular equilibria drove further aggregation into larger particles, leading to the formation of electrostatically better-stabilized assemblies because the total surface needing to be stabilized was decreased. The negative ζ-potential originates from the excess dye molecules bound to the dye-dendrimer electrostatic assemblies via dye.dye interactions. The weakening of the ζ-potential upon irradiation suggests that the over-stoichiometric dye ions were less strongly associated with the electrostatic dye-dendrimer complexes. The dye-dye interactions, particularly, were weakened upon irradiation, as the *cis* isomer bound less strongly than the *trans* isomer (see below). This disruption of secondary interactions reduced the surface charge density of the assemblies, making it insufficient to stabilize the numerous smaller particles. Consequently, the particles aggregated to form larger, more stable assemblies, which achieved sufficient stabilization through their remaining charge. Up to this point, the findings and conclusions are in agreement with our previous publications on this type of system [12,19].

The smaller assemblies generally exhibited stronger ζ-potential values, as illustrated in Figure 6b, providing insight into the relationship between the ζ-potential and particle size. Before irradiation, the assemblies were characterized by relatively small particle sizes (*R_H_* < 50 nm), as determined by DLS and exibited strongly negative ζ-potential values. In contrast, the larger assemblies that formed post-irradiation displayed smaller ζ-potential values.

Following irradiation, the reduction in the ζ-potential allowed the particles to come into closer proximity, leading to the formation of larger assemblies. To further interpret these results, the effective charge density (*σ*_eff_) was analyzed using Equation (S1), as shown in Figure 6c for the samples with monomodal size distributions.

Upon irradiation, a decrease in the charge density was observed, caused by the decrease in dye–dye interactions. This reduction was associated with the isomerization of the dye molecules to their *cis* form, which weakened the dipole–dipole interactions. Remarkably, the charge density was almost constant, with a characteristic value before and another one after the irradiation independent of the dendrimer generation. Thus, these experiments identified the charge density as a key parameter controlling the particle size. The molecular interactions determined a characteristic effective surface charge density, which, again, was realized by adopting a certain particle size and shape.

As a preferred effective surface charge density of the self-assembled nano-objects was evidently realized, both before and after irradiation (Figure 6b), rather than a specific ζ-potential or effective particle charge (Figure 6a and Figure S12c,d), it can be concluded that an adjustment in the particle morphology is needed to realize this effective surface charge density. Given that on a molecular level, based on association equilibria (see below), a certain degree of dye binding, and thereby a charge excess, occurred, while at the same time the building blocks occupied certain volumes, it is likely that certain effective charge density/volume scenarios can only be realized by non-spherical shapes. As visible in Figure S12b, the nano-object aspect ratio increases with its particle size, indicating the need of a surface area increase per particle. Further discussion will follow below.

### 3.3. Elucidating Interactions Before and After Irradiation by UV–vis Spectroscopy

**Figure 7:**
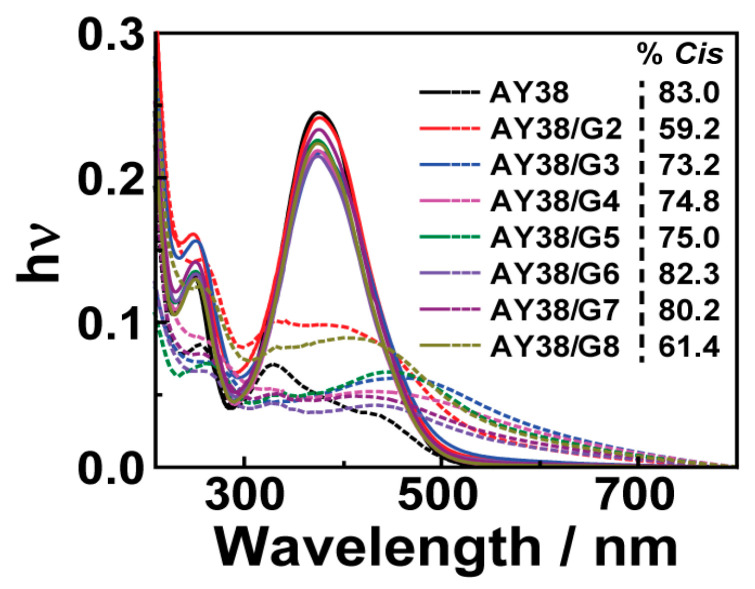
Effect of photoirradiation on dendrimer–dye assemblies at *l_c_* = 1.5 as analyzed by UV-vis spectroscopy. Straight lines: data before irradiation; dashed lines: after irradiation. The percentage of *cis* dye is given in the legend. Each color represents a UV-Vis spectrum of a different dye-dendrimer sample with the dendtrimer generation given in the plot.

In addition to the nano-structural (colloidal) characterization of the dye–dendrimer assemblies, molecular alterations arising from the dendrimer generation and photoisomerization were examined by UV–vis spectroscopy. As observed in the previous studies, the structural change was triggered by the *trans–cis* isomerization of the azo dye [12,20]. Before irradiation, the more stable *trans* isomer was in the majority (>90%), which was converted to the *cis* isomer at about 83% after 20 min of light irradiation with a *λ* = 365 nm UV lamp. In the dye–dendrimer assemblies, the spectral changes were analogous to the pure dye spectra before irradiation without any shifts, as shown in Figure 7.

The stability of the over-stoichiometric samples, as well as the negative ζ-potential of the assemblies, showed the involvement of secondary interactions among the dye molecules. The UV-vis spectra of the AY38 dye-dendrimer assemblies did not exhibit significant changes compared to the free dye, indicating that these secondary interactions were not dominated by π-π bonding. Such bonding would typically produce noticeable effects on the absorbance spectra of azo dyes. Instead, the origin of these interactions appears to be dipole-dipole interactions [19]. The *trans* dye molecules possessed a substantially higher dipole moment (27.96 Debye) compared to their *cis* counterparts (12.96 Debye), which increased the propensity for dye–dye interactions among the *trans* dye molecules as well as the strength of their mutual interactions. These interactions enhanced the binding capabilities of the dye molecules within the electrostatic assemblies [64]. Dipole-dipole interactions were particularly pronounced in the higher-generation dendrimers (≥G5), where the less flexible amino groups limited full dye-dendrimer binding, thereby promoting the formation of secondary dipole-dipole interactions.

The UV-vis and DLS measurements of the dye in water (without dendrimers) revealed no signs of aggregation [19]. This lack of aggregation highlights that the mutual dye–dye interactions only arose when the anionic dye solution was combined with the positively charged dendrimers. The electrostatic binding between the dye and dendrimers was more long-ranged and caused the “primary” aggregation. When associating, a dye preferably binds adjacently to another dye, undergoing both attractive dendrimer-dye ionic and “secondary” attractive dye-dye dipole-diploe interactions. These secondary dipole-dipole interactions among the dye molecules subsequently drive the formation of larger aggregates.

The UV-vis spectroscopy enabled the quantification of the *trans* and *cis* dye species in the samples [19]. Upon irradiation, the percentage of *cis* isomers increased, with its extent varying across the different dendrimer generations. The G2 dye assemblies exhibit the lowest *cis* content (≈59%), which progressively increased through the G3, G4, and G5 dendrimer samples, reaching its peak with the G6 (≈82%) and G7 (≈80%) assemblies before decreasing again in the G8 assemblies. This behavior is likely due to photoprotective effects observed in both the low- and high-generation dendrimer samples, driven by the predominance of interactions that hindered the isomerization. In the low-generation dendrimers, the flexible dendrimer structures favored electrostatic interactions, and the dye molecules strongly bound electrostatically, which partially hindered the isomerization of the *trans* dye molecules into the *cis* form.

On the other hand, as discussed, the higher-generation dendrimers were characterized by less flexible, sterically hindered architectures, causing an association with expressed dye-dye interactions. The steric conditions limited the dye binding, such that the attachment of only one sulfonate group of AY38 to the protonated amino groups and surplus dye ions occurred via dipole–dipole interactions. This again limited the dye isomerization. Hence, the largest *cis* amount existed with the medium generations.

**Figure 8:**
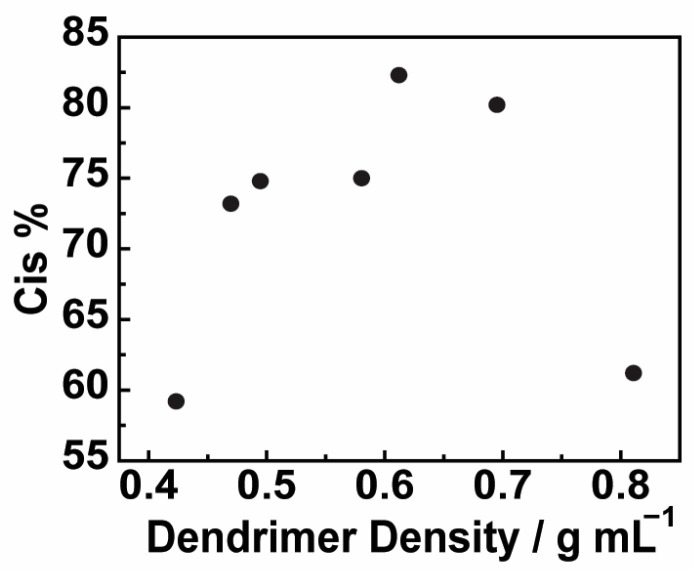
Relation between the percentage of *cis* isomers present in the dendrimer-dye samples after irradiation (*cis* %) and the average dendrimer density for the different dendrimer generations.

Figure 8 further considers the described scenario on a quantitative basis, displaying the dependence of the percentage of *cis* isomers present in the solution as function of the “dendrimer density”. Here, the average density of the dendrimer molecules was approximated using their molar mass and the volume of a sphere with the dendrimer extension (using the dendrimer diameter given by the supplier, which is accordance with the *R_H_* values determined by DLS (Figure S1, Table S1) and the radii of gyration from molecular dynamics simulations carried out by Maiti et al. [42]).

Interestingly, the total quantity of *cis* isomers in the solution (excluding G8) appeared to correlate directly with the change in the assembly size upon irradiation, as shown in Table 1. Similar to the highest *cis* percentage, the percentage change in size was also the largest in the G6 and G7 dendrimer assemblies. In a very different context, a comparable relationship between the size change and *cis* isomer content was previously reported by Willerich et al., who varied the *cis* amount in the solution by altering the irradiation wavelength [20]. In the G6 and G7 dendrimer assemblies, the dye molecules partially interacted with the tertiary amino group charges but also possibly predominantly bound to the primary amino groups (RNH_3_^+^) due to greater accessibility. The surplus dye ions engaged in secondary dipole–dipole interactions. This balance of interactions likely resulted in the dye ions binding to primary the amine charges of the G6 and G7 dendrimers via one sulfonate group (RSO_3_^−^), while the second sulfonate group remained unbound in solution. This flexibility allowed the unbound dye molecules to isomerize more easily, explaining the higher isomerization percentages observed in these assemblies.

From a molecular perspective, *cis* isomers, as previously described, possess a lower dipole moment compared to their *trans* counterparts and are less effective at forming strong dipole-dipole interactions between dye molecules [19]. Consequently, as the proportion of *cis* isomers increased under light irradiation, the weakened secondary interactions led to their reorganization into larger, again stable electrostatic assemblies.

The G8 PAMAM dendrimers exhibit the highest density and lowest flexibility among the studied generations, which limited the dye molecules’ ability to interact with the tertiary amine charges (R_3_NH^+^) electrostatically. Consequently, the dye molecules preferentially interact with the more accessible primary amino charges (RNH_3_^+^), leaving surplus dye ions available for secondary dye–dye interactions, limiting the isomerization of the dye molecules into the *cis* state in the G8 dendrimer assemblies but promoting their interconnection into larger assemblies.

### 3.4. Thermodynamic Analysis of the Assembly Formation

**Figure 9:**
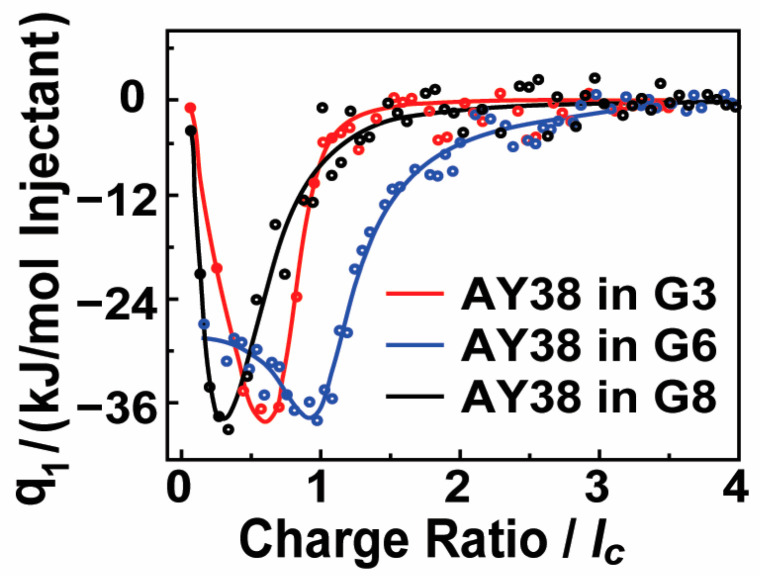
Isothermal titration calorimetry (ITC) to study the thermodynamics of the electrostatic dye–dendrimer assembly formation for varying dendrimer generations: AY38-G3, AY38-G6, and AY38-G8 (where the AY38 dye solution was titrated into the dendrimer solution). Here, *q*_1_ represents the energy produced by the system upon titrating per mole of azo dye into the solution and is plotted against the system’s charge ratios. Circles represent data, which were fitted by MicroCal Origin 7.0 software as shown by lines in the respective color.

ITC was performed to develop a fundamental understanding of the molecular interactions and thermodynamic parameters involved in the assembly formation. Recent findings indicate that the *trans* isomers of the dye molecules bind better over-stoichiometrically to the G4 dendrimer as compared to the *cis* isomers due to their different geometric structures. The *cis* isomers show almost no dye-dye interactions resulting from the low dipole moment in the *cis* state [19,64]. Previous studies have examined titrating the dendrimer solution (only G4) into the dye solution, where we observed a single-step reaction corresponding to the combination of the *trans* dye–dendrimer electrostatic interactions and the secondary dye-dye interactions [12,20,27]. Herein, for the first time, we elucidate the above-described dependence on the generation, where the dye-dye interactions were found to affect the assembly structures, necessitating a quantitative assessment of their impact. Figure 9 shows the titration of the dye AY38 into the dendrimer solutions with dendrimer generations of G3, G6, and G8, and the thermodynamic parameters of all the other measured generations are listed in Table 2, all before irradiation. As these measurements were performed before irradiation, the ITC results investigated only the *trans* isomers in the solution. The ITC curves in Figure 8 indicate a two-step reaction; the first step was found to be endothermic, characterized by an enthalpy change ΔH ≥ 6.7 kJ mol^−1^, which was accompanied by an increase in entropy (ΔS ≥ 1.3 kJ mol^−1^). In this first step, the excess of protonated amino groups of the dendrimer molecules led to electrostatic interactions, mainly due to the exchange of chloride ions (from DCl) with sulfonate groups from the AY38 molecules. During this process, the dye ions initially broke down the hydration shell around the dendrimer and counterion cloud, replacing the Cl^−^ ions with -SO_3_^−^ groups [65]. Therefore, this step resulted in a positive enthalpy change and entropy gain by the release of counterions, with an overall negative free energy change (ΔG ≤ −0.2 kJ mol^−1^) [66,67]. Additionally, the binding stoichiometry of the first step of the reaction is represented by N_1_, where N_1_ contributes less than 25% of the total number of available dendrimer sites. This suggests that the doubly charged azo dye molecules were only electrostatically bound per dendrimer. In this entropically driven first step, a decrease in ΔG upon increasing the dendrimer generation further hinted at the increment in the steric hindrance that primarily affected the electrostatic dye–dendrimer interactions, and this will be discussed in the following discussion section.

The second step was exothermic and enthalpically driven, with a binding enthalpy of ΔH ≤ −11.3 kJ mol^−1^ and a free energy change of ΔG ≤ −0.1 kJ mol^−1^. The second step corresponds to the combination of electrostatic and secondary dipole-dipole interactions [19]. The initial stage of the second step changed with the different dendrimer generations, but the step corresponded to a similar phenomenon for all generations. The binding sites of the divalent azo dye molecules per dendrimer increased as compared to the first step, where only electrostatic interactions were involved. In the second step, the charge capacity of the dendrimer was surpassed by an excess dye amount (*l_c_* > 1.0), leading to the above-mentioned stabilization of the negatively charged assemblies.

In the second step, the unique properties of the high-generation dendrimers became evident. The G6 dendrimer showed the most negative value of ΔH (compared to the other generations), showing that the assembly process was the most exothermic in the AY38/G6 system. This was due to the more significant number of terminal groups in the high-generation dendrimers available for binding with the dye molecules, which led to a more substantial number of favorable interactions, resulting in a decrease in free energy. However, the dye ions could only partially bind (one of the two binding sites) to the high-generation dendrimer molecules due to their strong steric hindrance, as mentioned above.

## 4. Discussion

In examining the generation dependence of dendrimer-dye assemblies prior to irradiation, up to the G6 dendrimer, no significant variation in the assembly size was observed with increasing the dendrimer generation. A decrease in size was noted with further increasing the dendrimer generation. Evidently, the structural changes in the conformation of the dendrimer molecules with increasing the dendrimer generation could significantly influence the characteristics of the dye-dendrimer assemblies. The lower-generation dendrimers (<G4) exhibited star-like, open molecular structures, while the medium-generation dendrimers (G4 and G5) resembled small, cross-linked polymer spheres. At higher generations (G6, G7, and G8), the dendrimers adopted nearly spherical shapes, with increasingly crowded internal structures (higher average density) and reduced molecular flexibility.

**Figure 10:**
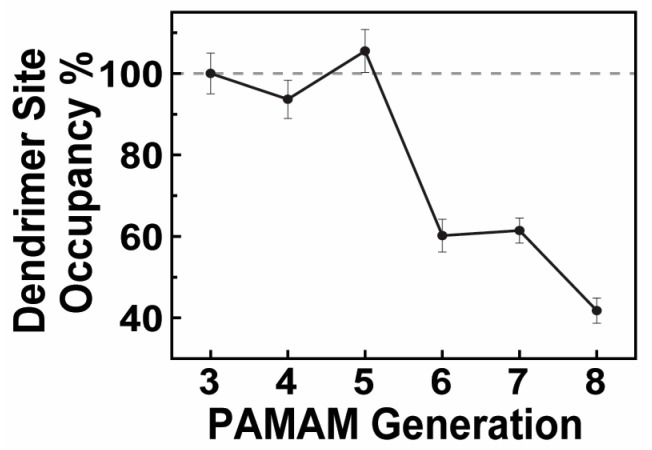
Dendrimer site occupancy of (primary + tertiary) dendrimer amino groups with Acid Yellow 38 dye sulfonate groups as a function of the dendrimer generation. (The value for the G4 PAMAM dendrimer was obtained from Willerich et al. [20].)

The percentage of dendrimer binding sites occupied by dye sulfonate groups per dendrimer molecule was quantitatively determined. It was calculated using the ratio of the total number of occupied sites (step 1 + step 2) determined via ITC to the theoretical number of available sites for each dendrimer generation. The results, presented in Figure 10, show that the lower-generation dendrimers (≤G5) exhibited near-complete site occupancy. This can be attributed to their high flexibility and thereby facile access of the dendrimer ionic groups for electrostatic interactions with the oppositely charged dye molecules.

For the G6 and G7 dendrimers, the site occupancy decreased to approximately 60%, and for the G8 dendrimers, it dropped further to around 40%. This finding can be attributed to the exponential increase in the number of amino groups with increasing the dendrimer generation. The AY38 molecules could not easily penetrate the increasingly crowded interior to a large extend, and dye stacks could not easily be formed in the inside of the higher-generation dendrimers; i.e., from the point of view of electrostatic interactions, a portion of the dye molecules was left free in the solution, which could then bind by dipole-dipole interactions to other (electrostatically bound) dye molecules. It is likely that most of the primary amines (which constitute 50% of the total amine charges) and only a limited number of the tertiary amines participated in interactions with the dye ions, as suggested by the UV–vis studies. The *cis* isomer percentage was also the highest for these mid-generation dendrimers, indicating that the bound dye ions remained readily accessible for isomerization. This suggests that the majority of the dye ions were attached to the outer branches of the dendrimers, and the dye–dye interactions did not dominate either, which minimized the photoprotective effect in these mid-generation dendrimers.

In the case of the G8 dendrimers, only about 40% of the sites were occupied by dye molecules, with additional dye molecules bound via dye.dye dipole interactions. These interactions again limited the isomerization process. Further, Figure S10 displays the dependency of the occupied dendrimer sites by dye sulfonate groups as a function of the average dendrimer density, confirming the above.

Thus, while increasing the dendrimer generation led to a rise in the number of amine groups and a potential for more electrostatic interactions with the dye molecules, the reduced flexibility of the higher-generation dendrimers restricted the dye-dendrimer interactions, promoting dye-dye interactions. This counterplay between an increased amine group availability and reduced structural flexibility is the origin of the observed generation-dependent site occupancy.

The change in the percentage of the hydrodynamic radius upon irradiation (*R_H_* %) from the light scattering results is plotted as a function of the dendrimer site occupancy in Figure S11a. Interestingly, the change upon irradiation was highest when a smaller number of dendrimer sites were occupied, which was also linked directly with the high *cis* %. This confirms that the dye molecules bound with the dendrimer ions via a single site and/or the dye molecules involved in only dipole-dipole interactions were the major contributors to the size change upon irradiation.

**Figure 11:**
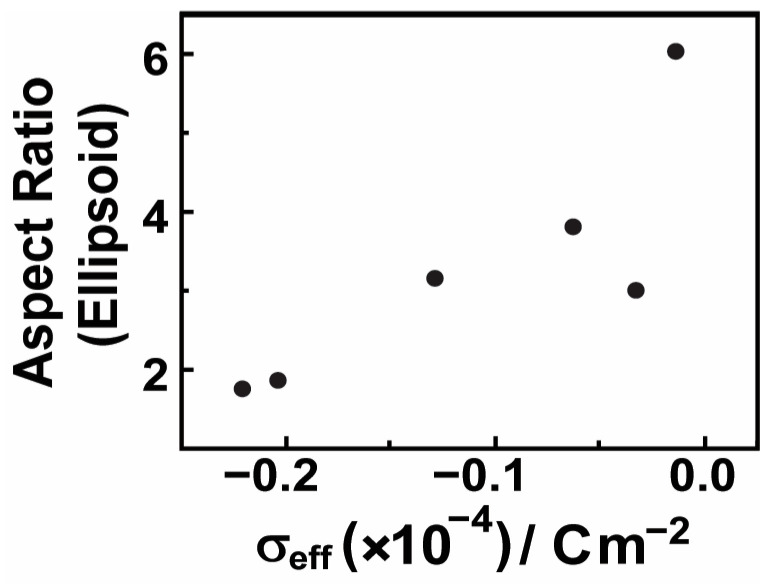
Nano-assembly aspect ratio obtained from SANS as function of the effective surface charge density.

As the SANS data revealed, the assembly shape also varied with the dendrimer generation and changed with photoirradiation. Upon irradiation, all the samples (except the G2 dendrimer) transitioned from spherical to ellipsoidal structures with different aspect ratios. Figure S11b represents the aspect ratio as a function of dendrimer site occupancy. Here, it is evident that the dendrimer molecules that interacted with a lower number of dye molecules formed more anisotropic structures, and the dendrimers with a high site occupancy formed less elongated ellipsoidal assemblies. It is likely that the structures with less occupied binding sites exhibited more remaining flexibility to adopt different particle shapes beyond globular structures. To understand why the elongated structures formed, it is interesting to consider the relationship between the particle shape and particle size. Remarkably, as Figure S12a,b shows, the aspect ratio of the ellipsoidal particles exhibited a direct relationship with the size of the assemblies rather than with the dendrimer generation. When the assembly size increased, the assemblies grew more anisotropically. It is likely that this was due to the charge stabilization and importance of the effective surface charge density, possibly in conjunction with other structural and density features. Certain effective surface charge densities per particle volume can rather be realized by anisotropic shapes. A direct connection between the surface charge density and the aspect ratio was observed, as shown in Figure 11. The aspect ratio increased when the effective charge density diminished (the negative value increased), i.e., the charge density was not only related to the particle size but also the particle shape. It is probable that an anisotropic shape on the nanoscale was adopted to avoid an energetically unfavorable charge-crowding (high surface charge density), as would occur for spherical particles. It is possible that the charge distribution played an additional role here. On a molecular level, this may also have been due to the anisotropy introduced through the dye dipole stacks within the structure, in addition to the above-mentioned nanoscale consideration.

## 5. Conclusions

In summary, we demonstrated an approach to control the size and shape of well-defined electrostatically self-assembled nanoparticles in an aqueous solution using different dendrimer generations and the dye AY38, as well as using UV light irradiation as a trigger to change the assembly structures. A complex scenario was found, where smaller assemblies were observed for small and large dendrimer generations and larger assemblies were observed for medium generations. On a molecular level, it was observed that the amount of *cis* dye depended on the dendrimer generation. We established a direct relationship between the amount of *cis* dye and the change in the size of the nanoparticles upon irradiation and elucidated the key role of the effective charge density in the size and shape control. In particular, the dendrimer generation encoded the number of dye molecules that interacted electrostatically, where electrostatics was more dominant at low generations, while secondary dipole–dipole interactions dominated at higher dendrimer generations, which is again what determines the degree of the photoprotective effect and thereby the amount of *cis* dye. This relationship may be used to predict the size increase by calculating the amount of *trans–cis* isomers in the solution based on UV-vis measurements only. Despite variations in the thermodynamic parameters (H, S, G, and K) with changing the dendrimer generation, the overall thermodynamics of these assemblies remained consistent, highlighting the robustness of the self-assembly process.

While dendrimers of different generations represent ideal model systems in this regard, the important role of molecular flexibility and charged group availability for association can be expected to be transferable to other linear and cross-linked polymer structures. As we have demonstrated a general association concept based on charges rather than specifically designed (and synthesized) binding motifs, the fundamental results will be usable in a broad field. Hence, these results are expected to be widely transferable within the versatile field of electrostatic self-assembly, combining ionic and secondary (here, dipole–dipole) interactions. Therefore, it is a potentially powerful and versatile method for producing desired assemblies with switchable sizes and shapes that could be used in various future applications.

## Figures and Tables

**Table 1:** Relationship between the percentage of cis dye in the sample (cis %) and the change in the aggregate size (*R_H_* %) upon irradiation for dendrimer–dye assemblies with PAMAM dendrimers of different generations.

PAMAM Generation	*cis*%	*R_H_*%
G2	59.2	411
G3	73.2	808
G4	74.8	461
G5	75.0	478
G6	82.3	1524
G7	80.2	1318
G8	61.2	1098

**Table 2:** ITC results of dye-dendrimer systems. AY38 was titrated into the dendrimer solution. Data are described with a two-site model (error bars ΔH: 3–5%, ΔG: 10–20%, N: 5–10%).

PAMAM Generation	G3	G5	G6	G7	G8
N_1_/sites	15	51	68	186	256
ΔH_1_ per site/kJ mol^−1^	89.2	6.7	30.3	58.9	28.4
ΔS_1_/kJ mol^−1^	4.4	1.3	69,036	367.7	24.5
ΔG_1_ per site/ kJ mol^−1^	−2.3	−1.1	−0.8	−0.3	−0.2
N_2_/sites	47	217	239	442	599
ΔH_2_ per site/kJ mol^−1^	−11.3	−34.6	−79.3	−43.1	−32.4
ΔS_2_/kJ mol^−1^	−1.6	−24.8	−63.2	−63.6	−64.8
ΔG_2_ per site/kJ mol^−1^	−1.1	−0.3	−0.2	−0.1	−0.1

## Data Availability

The data presented are included in this study. SANS data will be available at https://doi.ill.fr/10.5291/ILL-DATA.9-12-662, accessed on 30 November 2024.

## Supplementary Materials

The following supporting information can be downloaded at: https://www.mdpi.com/article/10.3390/polym17020170/s1, Figure S1: Multiangle DLS for the dendrimers of different dendrimer generations in D_2_O solution: electric field autocorrelation *g*_1_(*τ*) (open circular symbols), and distribution of relaxation times *A*(*τ*) (lines) at a scattering angle of *θ* = 90°: (**a**) G2, (**b**) G3, (**c**) G4, (**d**) G5, (**e**) G6, (**f**) G7, and (**g**) G8 (The *R_H_* values are obtained by extrapolation to a 0° angle); Figure S2: Multiangle DLS at a charge ratio (dye charges/dendrimer charges) of *l_c_* = 1.5 before (red) and after irradiation (blue): electric field autocorrelation *g*_1_(*τ*) (open circular symbols), and distribution of relaxation times *A*(*τ*) (lines) at a scattering angle of *θ* = 90° for different dendrimer generations: (**a**) G2, (**b**) G5, and (**c**) G7. (The *R_H_* values are obtained by extrapolation to a 0°angle and *σ* represents the particle size distribution at a 90° scattering angle, c_AY38_ = 1 × 10^−4^ mol L^−1^); Figure S3: Multiangle DLS of dendrimer-AY38 assemblies with high generation dendrimers (G6, G7, and G8) at *l_c_* = 1.5, before irradiation: electric field autocorrelation *g*_1_(*τ*) (open circular symbols), and distribution of relaxation times *A*(*τ*) (lines) at a scattering angle of *θ* = 70° (The *R_H_* values are obtained by extrapolation to a 0°angle and *σ* represents the particle size distribution at a 90° scattering angle, c_AY38_ = 1 × 10^−4^ mol L^−1^); Figure S4: Multiangle DLS of dendrimer-AY38 assemblies with G8 dendrimer at *l_c_* = 1.5, before irradiation: electric field autocorrelation *g*_1_(*τ*) (open circular symbols), and distribution of relaxation times *A*(*τ*) (lines) at multiple scattering angles of *θ*: (**a**) 40°, (**b**) 70°, (**b**) 120°, and (**b**) 150°; Figure S5: Diffusion coefficient, *D* as a function of the square of the scattering vector (*q*^2^) for monomodal assemblies in AY38/G3 system before irradiation, where *D* is extrapolated to *q*^2^ = 0 (= 0° scattering angle) to calculate the precise hydrodynamic radius of the nanoassemblies using Stokes-Einstein equation; Figure S6: Diffusion coefficient, *D* as a function of the square of the scattering vector (*q*^2^) for bimodal assemblies in AY38/G7 system before irradiation: (**a**) small assemblies (observed at all the scattering angles from *θ* = 30° to 150° with high-intensity peak size), (**b**) large aggregates (observed only at smaller scattering angles of *θ* < 90°, with low-intensity peak sizes), where *D* is extrapolated to *q*^2^ = 0 (= 0° scattering angle) to calculate the precise hydrodynamic radius of the nanoassemblies using Stokes-Einstein equation; Figure S7: Generation dependence of dendrimer-dye assemblies at a constant dendrimer number concentration. (1.0 × 10^−7^ mol L^−1^), for charge ratio *l_c_* = 2.0 (**a**) *R_H_* measured using multiangle DLS. The shaded area represents the polydispersity of the particle size. (**b**) Number concentration of the assemblies (approximated using the ratio of total scattering intensity and the volume (4/3 π *R_H_*^3^) of the assemblies) as a function of dendrimer generation. The open red symbols represent the second species of bimodal distributions; Figure S8: SLS-SANS of AY38/G2 system at *l_c_* = 1.5 before (red) and after irradiation (blue). The black lines represent the structural fits, corresponding to the particle shapes indicated in the plot; Figure S9: The effect of irradiation on aggregation number (the number of dendrimer units present in a particle) is calculated for dye-dendrimer assemblies at *l_c_* = 1.5 by varying dendrimer generations using (**a**) DLS and (**b**) SANS; Figure S10: Relation between the experimental site occupancy (of Acid Yellow 38 dye sulfonate groups with primary and tertiary amines in different PAMAM generations) with the respective dendrimer molecular density. (The site occupancy for G4 PAMAM dendrimer is obtained from Willerich et al. [20]). (The dendrimer density is calculated by dividing the molar mass of the dendrimer molecules by their molecular volume [43]; Figure S11: (**a**) Percentage difference in *R_H_* change upon irradiation and (**b**) Aspect ratio of the nano-assemblies after irradiation from SANS as function of dendrimer site occupancy; Figure S12: Further investigation of the structural control of the dendrimer-dye assemblies: (**a**) Aspect ratio of the nano-assemblies (from SANS) as function of dendrimer generation; (**b**) aspect ratio of the nano-assemblies (from SANS) as function of the hydrodynamic radius; (**c**) effective assembly charge (from the ζ-potential) as function of dendrimer generation; (**d**) effective assembly charge (from the ζ-potential) as function of the hydrodynamic radius.; Table S1: Comparison of theoretical (as given by supplier), experimental (as obtained from DLS), and *R_G_* values (observed by Maiti et al. [43] of dendrimer radius from different PAMAM generations.; Table S2: Structural parameters obtained from SANS for dendrimer-AY38 assemblies with and PAMAM dendrimers of different generations at *l_c_* = 1.5. All shapes were fitted using SasView 4.2.2 software with a high-quality fit (*χ*^2^ ≤ 3.0).
