# Supplementary material for: The Role of Light Irradiation and Dendrimer Generation in Directing Electrostatic Self-Assembly
Source: Polymers (Basel). 2025 Jan 11;17(2):170. doi: 10.3390/polym17020170 (PMC11769078; doi:10.3390/polym17020170)
Supplement: Supplementary file 1 [file polymers-17-00170-s001.zip › polymers-3375619-supplementary.pdf]

Supporting Information

The Role of Light-Irradiation and Dendrimer Generation in Directing Electrostatic Self-Assembly

Mohit Agarwal [1,2], Alexander Zika [1], Müge Yücel [1], Ralf Schweins [2], Joachim Kohlbrecher [3], and Franziska Gröhn[1,*]

Additional Results:

Figure S1 shows the sizes of the PAMAM dendrimers of different generations measured in D_2O as solvent. Multiangle DLS is used to determine the dendrimer size in solution. The concentration of the dendrimer solution was 1.0×10^{-6} mol L^{-1} for all samples. All dendrimer generations yield monomodal distributions. The resulting sizes are in agreement with the simulated values as given in Table S1.

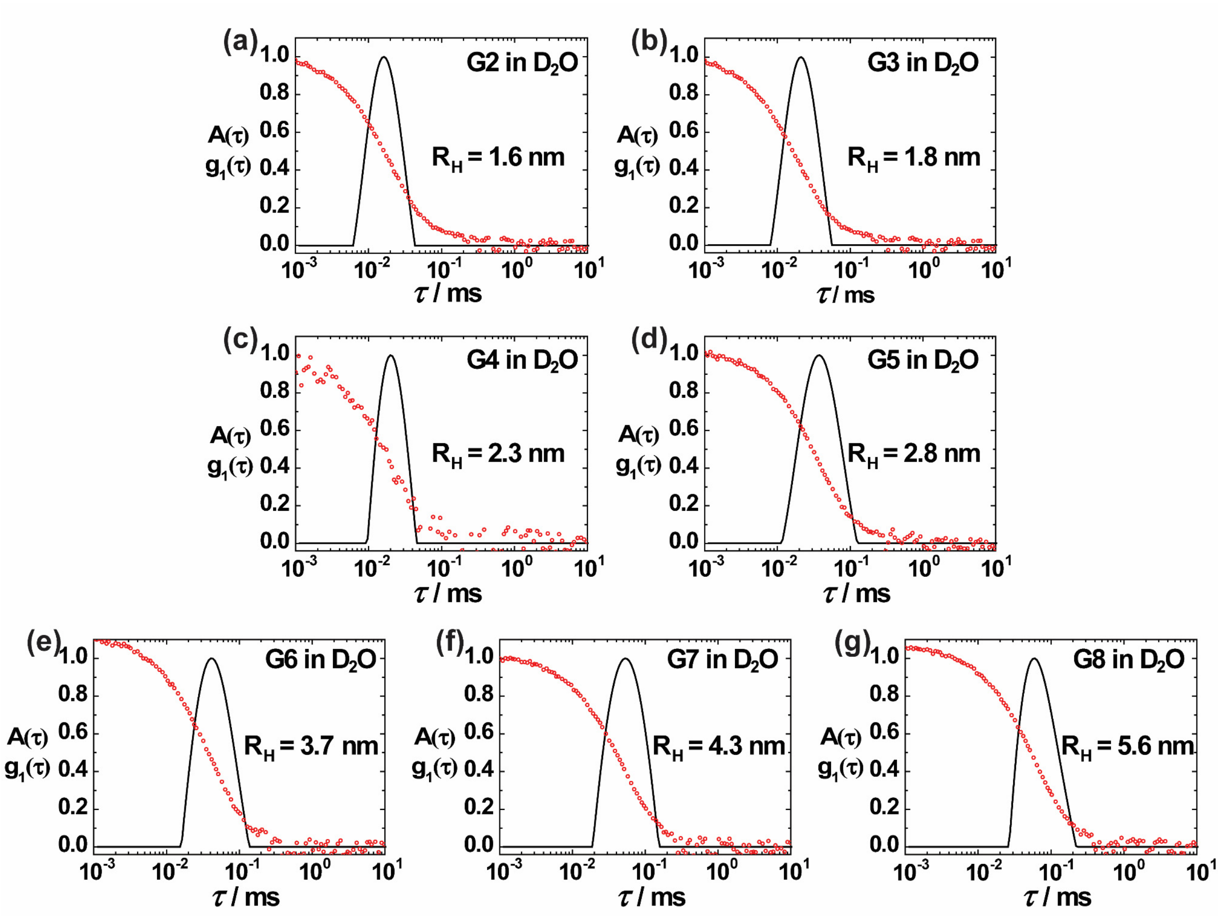

Figure S1 Multi-angle DLS for the dendrimers of different dendrimer generations in D_2O solution: electric field autocorrelation $g_1(\tau)$ (open circular symbols), and distribution of relaxation times $A(\tau)$ (lines) at a scattering angle of θ = 90°: **(a)** G2, **(b)** G3, **(c)** G4, **(d)** G5, **(e)** G6, **(f)** G7, and **(g)** G8 (The R_H values are obtained by extrapolation to a 0° angle).

Table S1 Comparison of theoretical (as given by supplier), experimental (as obtained from DLS), and R_G values (observed by *Maiti et al*. [43] of dendrimer radius from different PAMAM generations.

Dendrimer Generation	G2	G3	G4	G5	G6	G7	G8

**Theoretical radius/nm**	1.5	1.8	2.3	2.7	3.4	4.1	4.9
**Experimental Size(R_H)/nm**	1.6	1.8	2.3	2.8	3.7	4.3	5.6
**R_G/nm**	0.9	1.1	1.5	1.8	2.2	2.9	3.6

In Figure S2, electric field autocorrelation functions $g_1(\tau)$ are plotted with their corresponding relaxation of distribution times $A(\tau)$ at a 90° angle obtained from the dynamic light scattering instrument for dye-dendrimer assemblies at l_c = 1.5. Different generations of PAMAM dendrimers (G2, G5, and G7) were used.

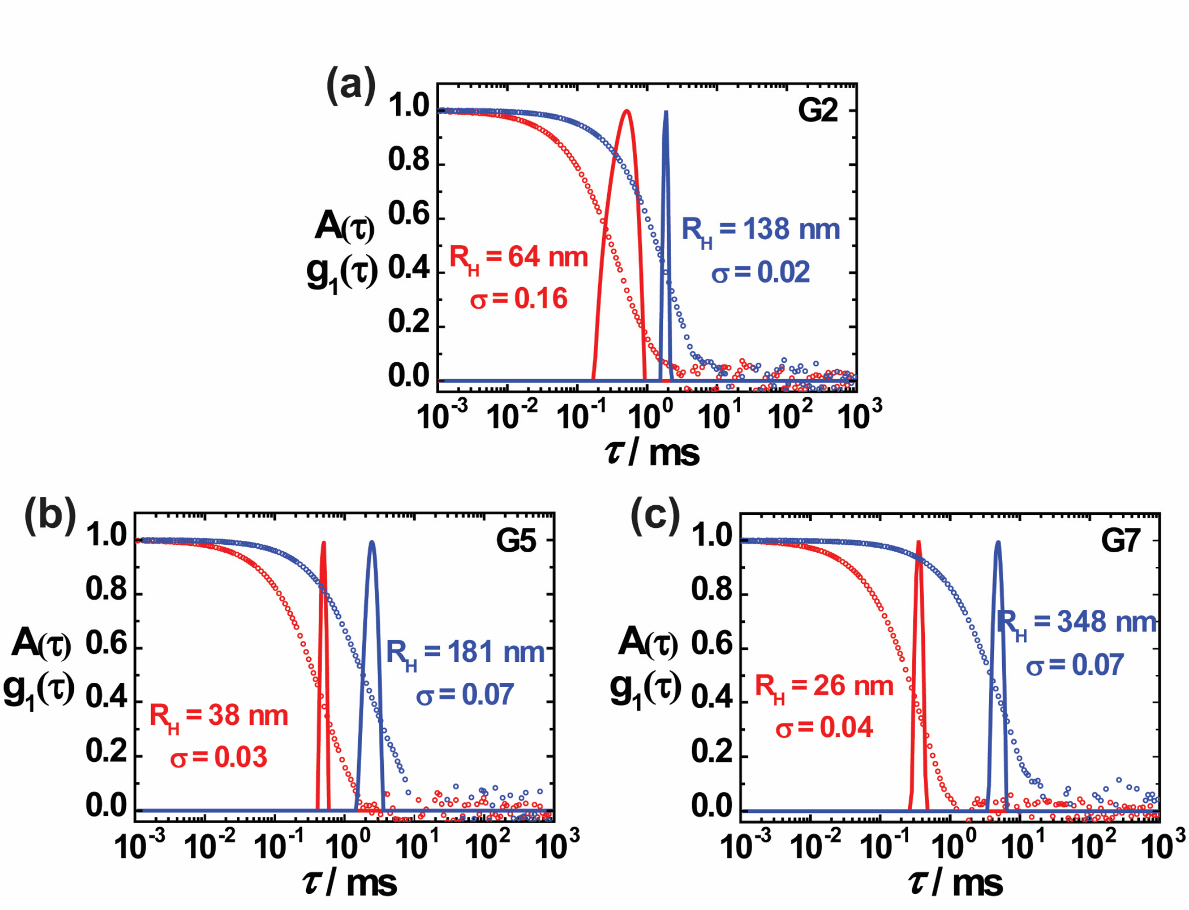

Figure S2 Multi-angle DLS at a charge ratio (dye charges/dendrimer charges) of l_c = 1.5 before (red) and after irradiation (blue): electric field autocorrelation $g_1(\tau)$ (open circular symbols), and

distribution of relaxation times $A(\tau)$ (lines) at a scattering angle of θ = 90° for different dendrimer generations: **(a)** G2, **(b)** G5, and **(c)** G7. (The R_H values are obtained by extrapolation to a 0°angle and σ represents the particle size distribution at a 90° scattering angle, c_{AY38} = 1 × 10^{-4} mol L^{-1}).

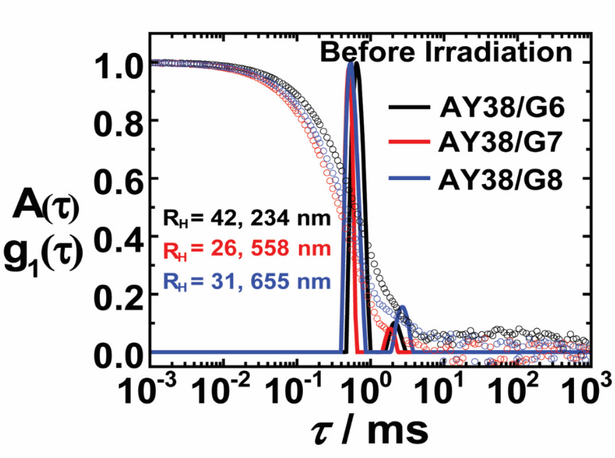

Figure S3 Multi-angle DLS of dendrimer-AY38 assemblies with high generation dendrimers (G6, G7, and G8) at l_c = 1.5, before irradiation: electric field autocorrelation $g_1(\tau)$ (open circular symbols), and distribution of relaxation times $A(\tau)$ (lines) at a scattering angle of θ = 70° (The R_H values are obtained by extrapolation to a 0°angle and σ represents the particle size distribution at a 90° scattering angle, c_{AY38} = 1 × 10^{-4} mol L^{-1}).

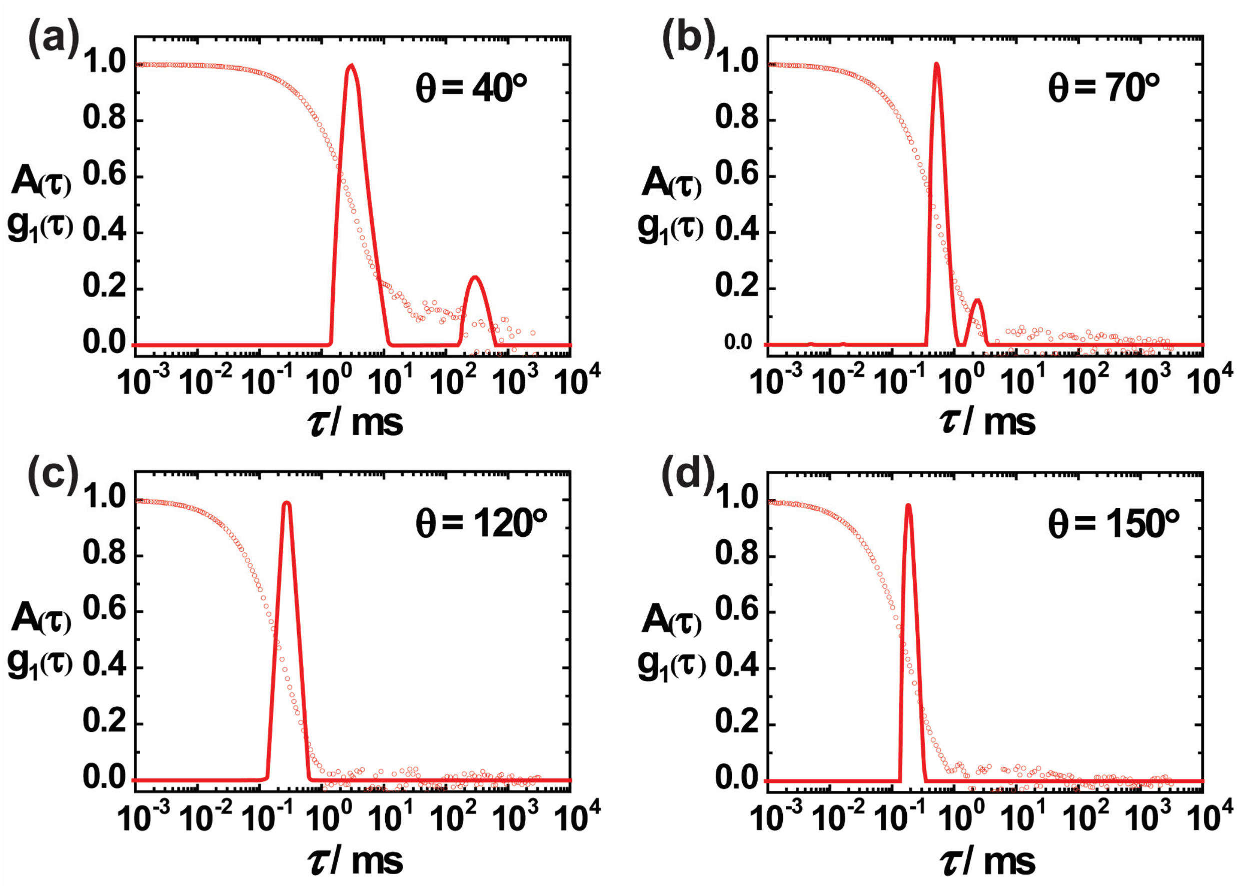

Figure S4 Multiangle DLS of dendrimer-AY38 assemblies with G8 dendrimer at l_c = 1.5, before irradiation: electric field autocorrelation $g_1(\tau)$ (open circular symbols), and distribution of relaxation times $A(\tau)$ (lines) at multiple scattering angles of θ: **(a)** 40°, **(b)** 70°, **(b)** 120°, and **(b)** 150°.

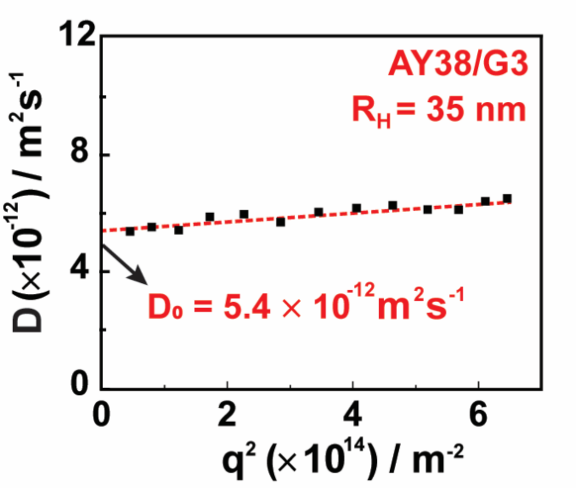

Figure S5 Diffusion coefficient, D as a function of the square of the scattering vector (q^2) for monomodal assemblies in AY38/G3 system before irradiation, where D is extrapolated to $q^2 = 0$ (= 0° scattering angle) to calculate the precise hydrodynamic radius of the nanoassemblies using Stokes-Einstein equation.

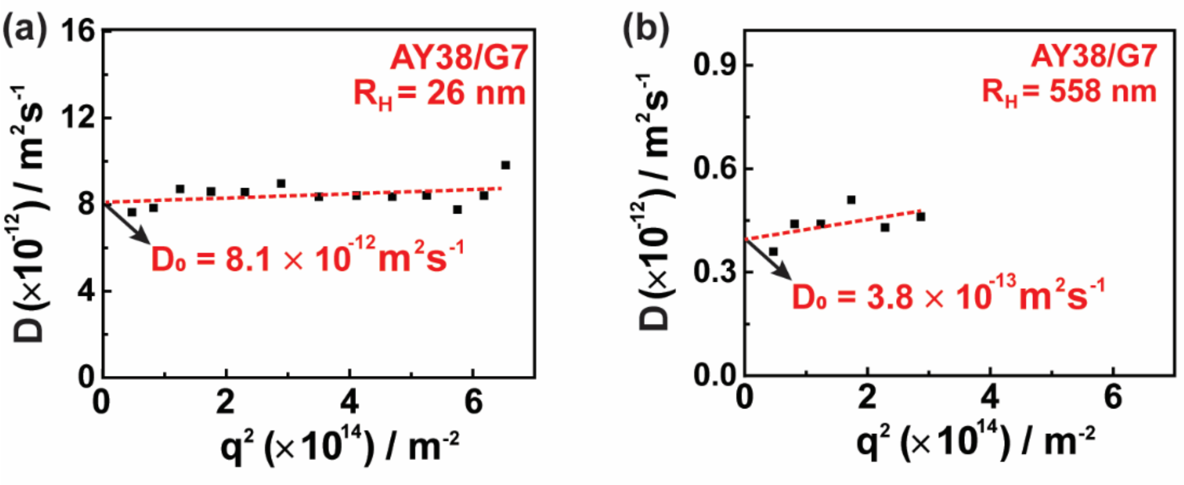

Figure S6 Diffusion coefficient, D as a function of the square of the scattering vector (q^2) for bimodal assemblies in AY38/G7 system before irradiation: **(a)** small assemblies (observed at all the scattering angles from θ = 30° to 150° with high-intensity peak size), **(b)** large aggregates (observed only at smaller scattering angles of θ < 90°, with low-intensity peak sizes), where D is extrapolated to $q^2 = 0$ (= 0° scattering angle) to calculate the precise hydrodynamic radius of the nanoassemblies using Stokes-Einstein equation.

A different study is conducted at l_c = 2.0, where the molar dendrimer concentration is kept constant at 1.0 × 10^{-7} mol L^{-1}. Different dendrimer generations and light irradiation show similar patterns as for the experiments with constant ass concentration (Figure S7). There is a difference in aggregate size (± 10 - 20%) observed in this case; still, the pattern remains identical, and the G6 dendrimer shows the most prominent size change upon irradiation. No significant size change occurs due to irradiation in the case of dendrimer excess, as reported in the literature [12].

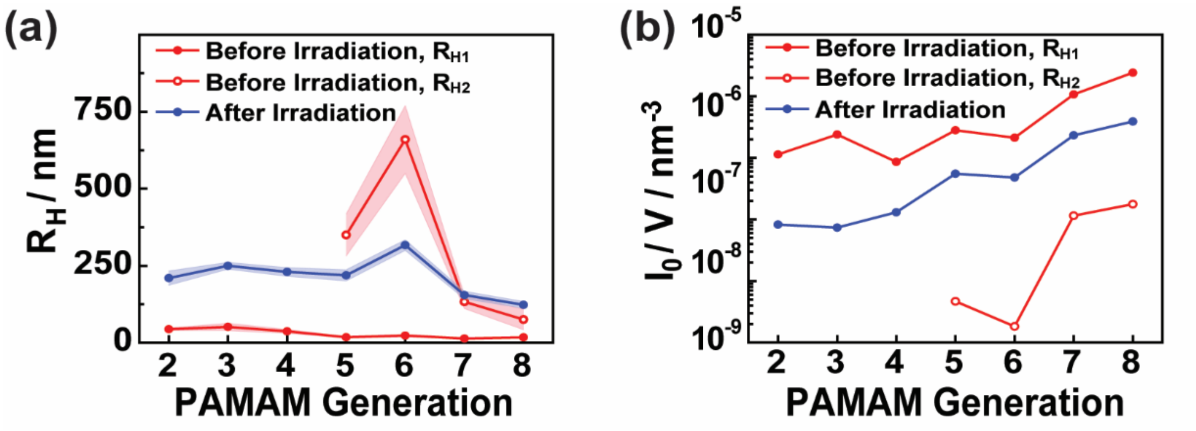

Figure S7 Generation dependence of dendrimer-dye assemblies at a constant dendrimer number concentration. (1.0 × 10^{-7} mol L^{-1}), for charge ratio l_c = 2.0 **(a)** R_H measured using multiangle DLS. The shaded area represents the polydispersity of the particle size. **(b)** Number concentration of the assemblies (approximated using the ratio of total scattering intensity and the volume ($4/3\ \pi\ R_H^3$) of the assemblies) as a function of dendrimer generation. The open red symbols represent the second species of bimodal distributions.

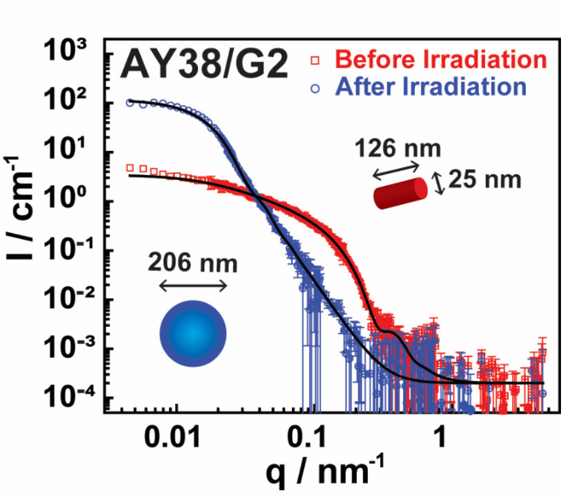

Figure S8 SLS-SANS of AY38/G2 system at I_c = 1.5 before (red) and after irradiation (blue). The black lines represent the structural fits, corresponding to the particle shapes indicated in the plot.

Table S2 Structural parameters obtained from SANS for dendrimer- AY38 assemblies with and PAMAM dendrimers of different generations at l_c = 1.5. All shapes were fitted using SasView 4.2.2 software with a high-quality fit ($\chi^2 \leq 3.0$).

System	Irradiation Condition	Shape	Cross section		Length /nm	Aspect Ratio
			R_{min} / nm	R_{maj} / nm		
**AY38 + G2**	Before	Cylinder	13 ± 2		126 ± 11	4.8
	After	Sphere	103 ± 6			1.0
**AY38 + G3**	Before	Sphere	26 ± 3			1.0
	After	Ellipsoid	91 ± 12	270 ± 22		2.9
**AY38 + G4**	Before	Sphere	29 ± 1			1.0
	After	Ellipsoid	92 ± 8	160 ± 14		1.7
**AY38 + G5**	Before	Sphere	283 ± 3			1.0
	After	Ellipsoid	95 ± 7	177 ± 15		1.8
**AY38 + G6**	Before	Sphere	30 ± 2			1.0
	After	Ellipsoid	100 ± 6	603 ± 48		6.0
**AY38 + G7**	Before	Sphere	20 ± 2			1.0
	After	Ellipsoid	113 ± 11	427 ± 34		3.8
**AY38 + G8**	Before	Sphere	19 ± 1			1.0
	After	Ellipsoid	90 ± 5	283 ± 24		3.1

The size of the assemblies varies depending on the dendrimer generations. Therefore, it is crucial to calculate the aggregation number of dendrimers within a single nanoparticle. From the results of light scattering, all the particles are assumed to be spherical, and the volume of the particles was calculated using the volume of a sphere. The aggregation number was calculated using the volume of the assembled particle divided by a sphere volume with a diameter of a single dendrimer molecule plus the extension of a bound dye molecule (Figure S9a). Further, the particle volumes were calculated from the SANS structural fit results; i.e. considering the particle shapes (Figure S9b) Both measurements

show the decay in aggregation number while increasing the charge ratio, which differs after irradiation.

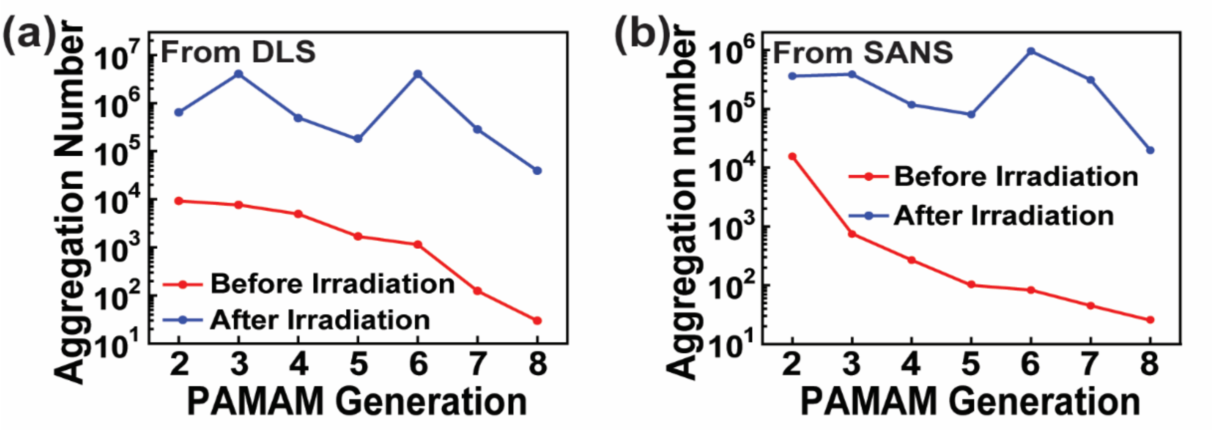

Figure S9 The effect of irradiation on aggregation number (the number of dendrimer units present in a particle) is calculated for dye-dendrimer assemblies at I_c = 1.5 by varying dendrimer generations using **(a)** DLS and **(b)** SANS.

To understand the electrostatic properties further, the effective surface charge density σ_{eff} and effective charge Q_{eff} are of interest in addition to the measured ζ-potential itself. To receive the σ_{eff}, the following equation is used:

$$\sigma_{eff} = \frac{Q_{eff}}{S_{assembly}} = \frac{4\pi\varepsilon\zeta R_{eff}}{4\pi R_{eff}^2} = \frac{\varepsilon\zeta}{R_{eff}} \approx \frac{\varepsilon\zeta}{R_H} \qquad \textbf{(S1)}$$

Where Q_{eff} is the effective surface charge, $S_{assembly}$ is the assembly surface, ε is the permittivity constant, and R_{eff} is the effective radius, approximated as the hydrodynamic radius, R_H. High σ_{eff} values refer to densely charged particles.

Further, the dendrimer site occupancy can be determines. The number of occupied binding sites, i.e. the sum of the two binding site numbers two-site model in ITC, is divided

by the total number of dendrimer binding sites theoretically available for dye interactions, i.e. the number of dendrimer amine groups.

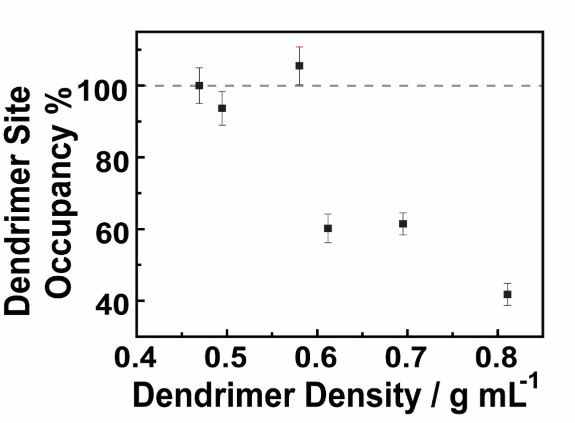

Figure S10 Relation between the experimental site occupancy (of Acid Yellow 38 dye sulfonate groups with primary and tertiary amines in different PAMAM generations) with the respective dendrimer molecular density. (The site occupancy for G4 PAMAM dendrimer is obtained from *Willerich et al.* [20]). (The dendrimer density is calculated by dividing the molar mass of the dendrimer molecules by their molecular volume [43]

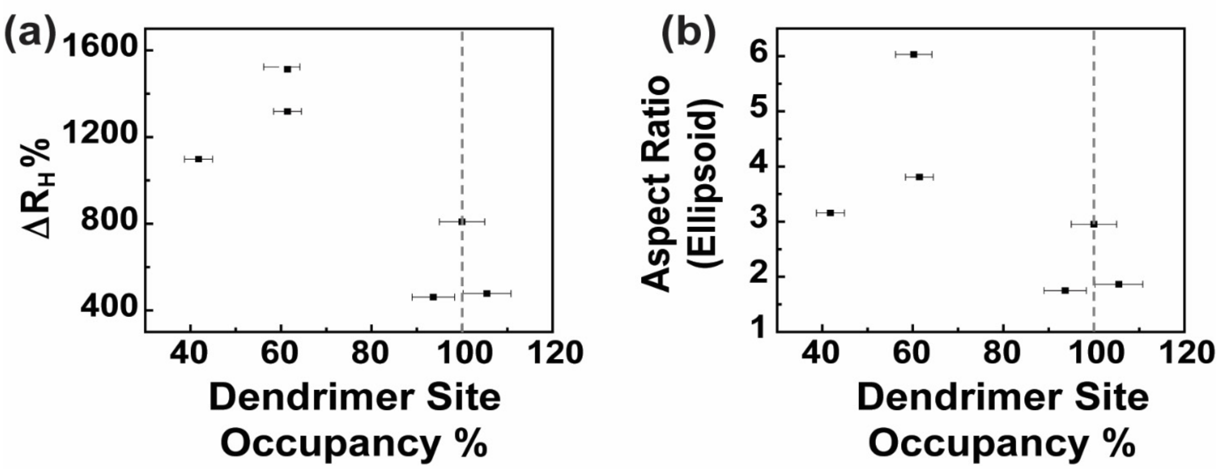

Figure S11 (a) Percentage difference in R_H change upon irradiation and **(b)** Aspect ratio of the nano-assemblies after irradiation from SANS as function of dendrimer site occupancy

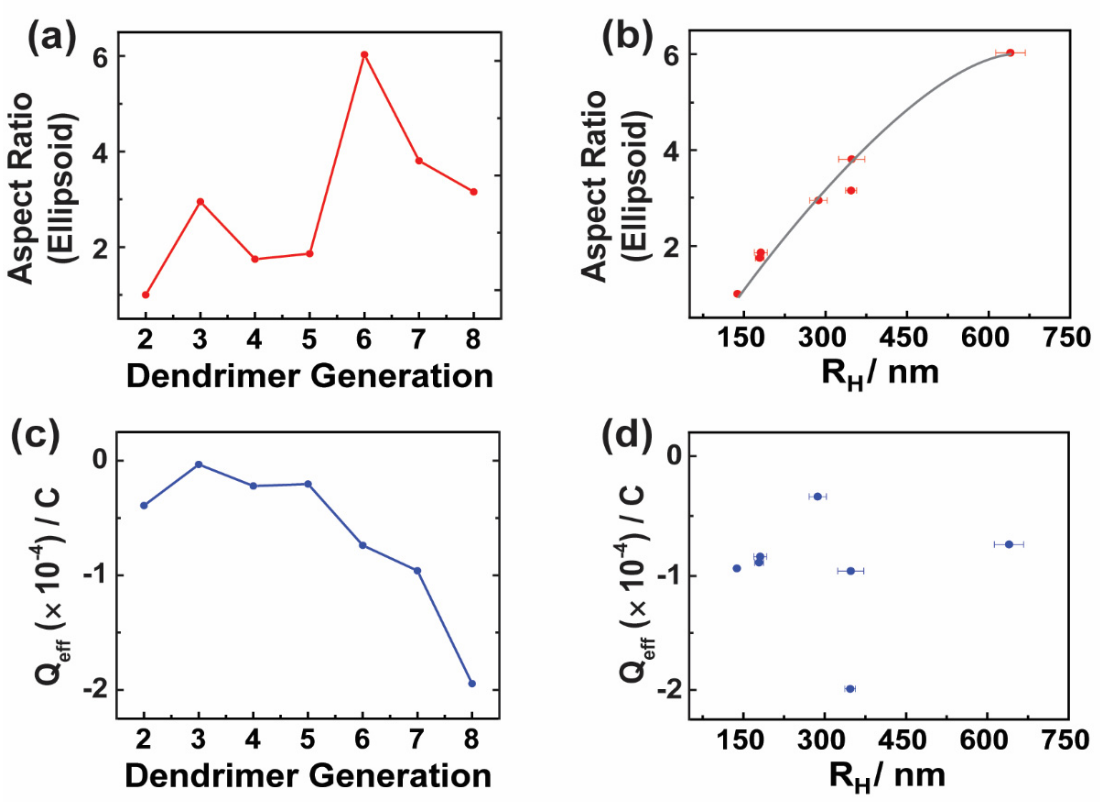

Figure S12 Further investigation of the structural control of the dendrimer-dye assemblies: **(a)** Aspect ratio of the nano-assemblies (from SANS) as function of dendrimer generation; **(b)** aspect ratio of the nano-assemblies (from SANS) as function of the hydrodynamic radius; **(c)** effective assembly charge (from the ζ-potential) as function of dendrimer generation; **(d)** effective assembly charge (from the ζ-potential) as function of the hydrodynamic radius.

References:

12. Willerich, I.; Gröhn, F. Photoswitchable Nanoassemblies by Electrostatic Self-Assembly. *Angew. Chemie - Int. Ed.* **2010**, *49*, 8104–8108, doi:10.1002/anie.201003271.

20. Willerich, I.; Gröhn, F. Thermodynamics of Photoresponsive Polyelectrolyte-Dye Assemblies with Irradiation Wavelength Triggered Particle Size. *Macromolecules* **2011**, *44*, 4452–4461, doi:10.1021/ma200538e.

43. Maiti, P.K.; Çağin, T.; Wang, G.; Goddard, W.A. Structure of PAMAM Dendrimers: Generations 1 through 11. *Macromolecules* **2004**, *37*, 6236–6254, doi:10.1021/ma035629b.
